# Prediction of Transmembrane Regions, Cholesterol, and Ganglioside Binding Sites in Amyloid-Forming Proteins Indicate Potential for Amyloid Pore Formation

**DOI:** 10.3389/fnmol.2021.619496

**Published:** 2021-02-10

**Authors:** Katja Venko, Marjana Novič, Veronika Stoka, Eva Žerovnik

**Affiliations:** ^1^Theory Department, National Institute of Chemistry, Ljubljana, Slovenia; ^2^Department of Biochemistry and Molecular and Structural Biology, Jožef Stefan Institute, Ljubljana, Slovenia

**Keywords:** amyloid-forming proteins, amyloidogenic regions, transmembrane regions, amino-acid sequence predictors, cholesterol and ganglioside binding motifs, amyloid pore

## Abstract

Besides amyloid fibrils, amyloid pores (APs) represent another mechanism of amyloid induced toxicity. Since hypothesis put forward by Arispe and collegues in 1993 that amyloid-beta makes ion-conducting channels and that Alzheimer's disease may be due to the toxic effect of these channels, many studies have confirmed that APs are formed by prefibrillar oligomers of amyloidogenic proteins and are a common source of cytotoxicity. The mechanism of pore formation is still not well-understood and the structure and imaging of APs in living cells remains an open issue. To get closer to understand AP formation we used predictive methods to assess the propensity of a set of 30 amyloid-forming proteins (AFPs) to form transmembrane channels. A range of amino-acid sequence tools were applied to predict AP domains of AFPs, and provided context on future experiments that are needed in order to contribute toward a deeper understanding of amyloid toxicity. In a set of 30 AFPs we predicted their amyloidogenic propensity, presence of transmembrane (TM) regions, and cholesterol (CBM) and ganglioside binding motifs (GBM), to which the oligomers likely bind. Noteworthy, all pathological AFPs share the presence of TM, CBM, and GBM regions, whereas the functional amyloids seem to show just one of these regions. For comparative purposes, we also analyzed a few examples of amyloid proteins that behave as biologically non-relevant AFPs. Based on the known experimental data on the β-amyloid and α-synuclein pore formation, we suggest that many AFPs have the potential for pore formation. Oligomerization and α-TM helix to β-TM strands transition on lipid rafts seem to be the common key events.

## Introduction

It is widely accepted and inherited cases confirm a notion that the major part of the pathology of neurodegenerative diseases is due to aberrant processes of protein misfolding and formation of amyloid fibrils by the amyloidogenic proteins concerned: α-synuclein in Parkinson's disease, β-amyloid (Aβ) in Alzheimer's disease, SOD1 and TDP-43 in amyotrophic lateral sclerosis, etc. Dobson (2002) discovered that these conformational transitions are not reserved to amyloidogenic proteins, but that under certain conditions all proteins can be converted into amyloid fibrils, even the very stable and α-helical myoglobin (Fandrich et al., 2001). However, the tendency to misfold and aggregate to amyloid at physiological pH and temperature is not the same for all proteins; certain proteins or their parts—after cleavage—are more susceptible to the formation of amyloid fibrils. Amyloidogenic proteins do not have common sequence motifs, but by comparing the protein sequences it can be predicted that some parts are hot spots that form a cross-β spine of amyloid-like fibrils (Nelson et al., 2005). The peptides, which are as short as hexapeptides, can form amyloid fibrils (Tenidis et al., 2000). From the molecular forces that determine the cross-β structure of the amyloid, the main chain hydrogen bonds, but also aromatic repetitive patterns (Gazit, 2007) seem to be of great importance, the latter probably undergoing Π stacking (Gazit, 2002, 2007; Reymer et al., 2014). The secondary structure in the native fold protein is important, but not directly correlated with the secondary structure of the amyloid fibrils. The over-prediction of α-helices compared to the X-ray structure derived α-helices indicates the propensity of α to β transition in the intermediate (Morillas et al., 2001), partially unfolded state and, for intrinsically disordered proteins, partially folded state.

The transition to amyloid fibrils is a reaction consisting of a lag, growth and plateau phases. The most common mechanism is nucleation via an oligomeric nucleus and the other spectrum is downhill polymerization (Žerovnik et al., 2011; Dovidchenko et al., 2014). In due course of amyloid fibrils formation the prefibrillar oligomers of different shapes can be formed; from rings as found at Aβ (Oxana, 2019), to globules, spheres, or stars. Some of these prefibrillar oligomers are on-pathway and determine the nucleus that assigns the lag phase, some are off-pathway. Some are benign some are toxic differing by subtle changes in conformation (Capitini et al., 2018; Sengupta and Udgaonkar, 2018). The toxic prefibrillar oligomers (Bucciantini et al., 2002; Leri et al., 2016) are thought to make pores into membranes, similar to antimicrobial peptides or bacterial toxins (Anderluh and Žerovnik, 2012; Last and Miranker, 2013). The “channel hypothesis” of AD is not new. It is based on electrophysiological measurements by the group of Arispe et al. (1993a,b); Arispe et al. (2014), Kawahara et al. (2000), and Diaz et al. (2009). Later, the same concept was increased to other amyloid proteins, among them α-synuclein (α-syn), by Lashuel et al. (2002) and Lashuel and Lansbury (2006). Amyloid pore (AP) formation (Kawahara et al., 2011; Di Scala et al., 2016; Kandel et al., 2017) is still not fully understood and has not been directly proven in living cells until recently (Jamasbi et al., 2018). Various *in vitro* studies on membrane vesicles, artificial lipid bilayers, and neuronal cell cultures were performed for Aβ and α-syn (Kawahara et al., 2000; Tsigelny et al., 2012; Chen et al., 2016; Di Scala et al., 2016; Kandel et al., 2017; Hannestad et al., 2020; Perissinotto et al., 2020). Recent, still *in vitro* study revealed the imaging of how α-syn forms the AP in membrane predominantly composed of anionic phospholipids, alike those making mitochondrial membranes. Since the interaction of neuronal α-syn with lipid membranes appears crucial in the context of Parkinson's disease, authors tried to explain the roles of different lipids in pathogenic protein aggregation and membrane disruption (Hannestad et al., 2020). Perissinotto et al. (2020) showed that metals (iron in particular) influence interaction of α-syn with lipid rafts. Kayed et al. (2020) has written a review on existence of endogenous oligomeric and multimeric species in α-synucleopathies. The association of α-syn with plasma membrane of hippocampal neurons was demonstrated to induce the formation of pore-like structures (Li et al., 2020). The analysis of Lee et al. (2017) has shown structure and conductance of oligomeric Aβ pores in a natural lipid membrane, which closely mimics the *in vivo* cellular environment. Recent studies also include interaction of Aβ with cellular membranes (Bode et al., 2017) and animal models (Julien et al., 2018), both confirming the hypothesis of membrane perforation. For example, in *C. elegans* the membrane repair response was turned on when Aβ was fed to animals (Julien et al., 2018).

Moreover, in last years the researchers have elucidated the X-ray crystallographic structures of oligomers derived from Aβ, α-synuclein, and β2-microglobulin (Kreutzer and Nowick, 2018). Out of these three amyloidogenic peptides/proteins, the Aβ β-hairpin mimics have provided the most insight into amyloid oligomers. Study has revealed a new mode of self-assembly, where three Aβ β-hairpin mimics assemble to form a triangular trimer which can pack together with other triangular trimers to form higher-order oligomers (hexamers and dodecamers). These higher-order oligomers can form annular pore-like assemblies and exhibit toxicity toward neuronally derived cells (Kreutzer and Nowick, 2018). Specific pore-forming β-barrel oligomers of Aβ42 in DPC micelle conditions were reported also by Serra-Batiste et al. (2016). Recently, another atomic level structures of β-sheet pore-forming Aβ(1-42) oligomers were obtained by nuclear magnetic resonance (NMR) and mass spectrometry (MS), and a mechanism for membrane disruption based on electrophysiology and simulation studies in membranes was provided (Ciudad et al., 2020). These structural findings are significant and address the gap in understanding the molecular basis of amyloid diseases.

Various methods have been developed to calculate the propensity to form amyloid fibrils, such as AGGRESCAN, AGGRESCAN3D, TANGO, WALTZ, etc. The method AGGRESCAN3D (Pujols et al., 2018) takes into account the tertiary structure of proteins apart from their sequence. The overview of the available programs is described in the review paper of Pallarés and Ventura (2019). A preliminary screening of amyloidogenic sequence fragments can be performed with the RFAmy predictor (http://server.malab.cn/RFAmyloid) (Niu et al., 2018) and the AmyPro database (https://amypro.net) (Varadi et al., 2018). This database includes pathogenic amyloids as well as prions and functional amyloids, and allows users to screen their sequences against the entire collection of validated amyloidogenic sequence fragments. Further, AmyPred2 (Tsolis et al., 2013) (http://aias.biol.uoa.gr/AMYLPRED2) shows a CONSENSUS result of many methods. Previously, this program was successfully used to predict amyloid-prone regions in human stefin B wild-type and proline mutants (Hasanbasic et al., 2019). In this study a set of 30 potentially AFPs was selected and the amyloid-fibril propensity was calculated using various tools (Table 1).

**Table 1:** The list of 30 studied amyloid-forming proteins.

**ID**	**Protein name**	**UniProtKB ID**	**Amyloid category**	**RFAmy probability**^**a**^	**Amyloidogenic regions AmyPRO database**^**b**^ **and AmylPred2^c^**
1	β-amyloid	P05067	Pathological	0.731	11–42^b^, 16–21^c^, 30–42^c^
2	α-synuclein	P37840	Pathological	0.783	35–81^b^, 36–42^c^, 52–65^c^
3	Prion protein	P04156	Pathological	0.849	84–125^b^, ^c^, 148–171^b^, ^c^, 194–216^b^, ^c^, 223–231^b^
4	Tau protein	P10636	Pathological	0.842	275–280^b^, ^c^, 302–329^b^, 369–370^c^, 373–373^c^, 397–402^c^
5	β-2 microglobulin	P61769	Pathological	0.788	21–41^b^, ^c^, 54–71^b^, ^c^, 83–89^b^, ^c^, 91–96^b^
6	Cystatin C	P01034	Pathological	0.848	47–51^b^, ^c^, 56–65^b^, ^c^, 95–104^b^, ^c^
7	Transthyretin	P02766	Pathological	0.864	10–20^b^, ^c^, 26–34^c^, 91–96^b^, ^c^, 105–115^b^, ^c^, 119–124^b^, ^c^
8	Lysozyme C	P61626	Pathological	0.783	5–14^b^, 25–34^b^, ^c^, 56–61^b^, ^c^, 76–84^c^, 107–112^c^
9	IAPP-amylin	P10997	Pathological	0.807	11–37^b^, 13–24^c^
10	Calcitonin	P01258	Pathological	0.704	6–11^b^, ^c^, 15–20^b^
11	Prolactin	P01236	Pathological	0.785	7–34^b^, 21–31^c^, 43–57^b^, 80–89^c^, 95–101^c^, 130–137^c^, 167–174^c^, 187–195^c^
12	Insulin	P01308	Pathological	0.733	13–18^b^, ^c^, 18–25^c^, 32–38^b^, ^c^, 44–48^c^
13	TDP-43	Q13148	Pathological	0.775	26–33^c^, 55–60^c^, 69–76^c^, 105–111^c^, 123–135^c^, 148–153^c^, 216–221^c^, 225–234^c^, 247–256^c^, 381–407^b^
14	Superoxide dismutase 1	P00441	Pathological	0.761	4–7^c^, 12–23^b^, 101–107^b^, ^c^, 112–120^c^, 147–153^b^, ^c^
15	Stefin B (cystatin B)	P04080	Pathological	0.780	39–59^b^, ^c^, 64–71^b^, ^c^, 80–87^b^, 95–98^b^
16	α-crystallin B chain	P02511	Pathological	0.701	26–29^c^, 70–84^c^, 91–98^c^
17	α-1-antichymo-trypsin	P01011	Pathological	0.068	31–42^c^, 50–66^c^, 179–192^c^, 216–228^c^, 238–243^c^, 250–255^c^, 287–290^c^, 304–308^c^, 333–340^c^, 355–364^c^, 366–369^c^, 376–382^c^, 384–392^c^
18	Stefin A (cystatin A)	P01040	Biologically non-relevant	0.806	46–57^c^, 65–70^c^, 81–85^c^
19	Myoglobin	P02144	Biologically non-relevant	0.816	2–30^b^, ^c^, 68–73^c^, 102–119^b^, ^c^
20	α-phosphatidyl inositol 3-kinase	P27986	Biologically non-relevant	0.841	23–28^b^, 38–44^c^, 74–79^c^
21	Cathelicidin	P49913	Pathological	0.844	5–6^c^
22	Secretin	P09683	Negative control	0.859	21–26^c^
23	Corticoliberin	P06850	Functional	0.334	1–40^b^, ^c^
24	GIP—gastric inhibitory polypeptide	P09681	Functional	0.864	1–43^b^, 23–28^c^
25	Urocortin	P55089	Functional	0.839	1–40^b^, ^c^
26	α-crystallin A chain	P02489	Pathological	0.783	66–80^b^, 23–27^c^, 37–45^c^, 50–56^c^, 70–78^c^
27	Obestatin	Q9UBU3	Functional	0.689	1–23^b^
28	Glucagon	P01275	Functional	0.699	1–10^b^, 22–27^c^
29	Defensin-6	Q01524	Functional	0.713	1–32^b^, 21–31^c^
30	β-endorphin	P01189	Functional	0.704	1–31^b^, 14–23^c^

a *RFAmy predictor (http://server.malab.cn/RFAmyloid)*.

b *AmyPRO database (https://amypro.net)*.

c *AmylPred2 predictor (http://aias.biol.uoa.gr/AMYLPRED2)*.

In the interaction of prefibrillar oligomers with phospholipid membranes, the lipid rafts, i.e., the microdomains of membranes rich in gangliosides and cholesterol, play an important role (Jang et al., 2009, 2013; Di Scala et al., 2016; Kandel et al., 2019). There are some parallels to the entry of virus particles (Yahi and Fantini, 2014). For example, the spike protein of the coronavirus SARS-CoV-2 in the S2-part has a motif that binds to lipid rafts and thus enables the S1-part to attach and interact with the ACE2-receptor (Fantini et al., 2020). Of interest, the SARS coronavirus-(SARS CoV-1) protein E (E for envelope) was shown to form cation-selective membrane channels (Wilson et al., 2004; Verdiá-Báguena et al., 2012). The SARS-CoV-2 protein E thus likely functions as a “viroporin,” but also may have an important function in the infection process and subsequent inflammation (Pacheco et al., 2015).

**Figure 1:**
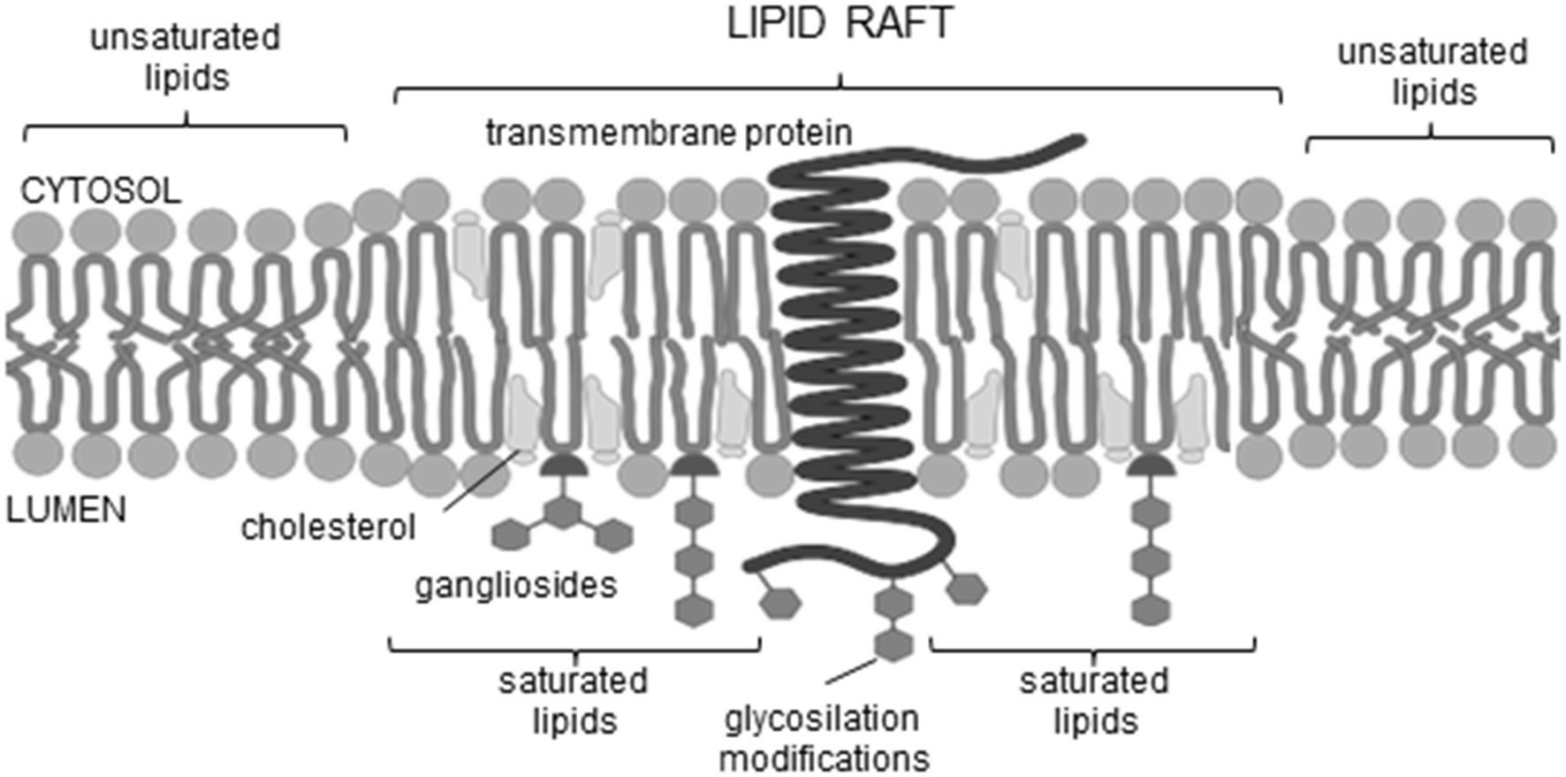
Lipid raft scheme.

Therefore, the main focus of our study was to determine in the set of 30 AFPs the domains that could be crucial for AP formation [TM regions, ganglioside (GBM) and cholesterol binding motifs (CBM)]. In this regard, several publicly available tools were used to assess whether the proteins under investigation have TM regions, either α-helices or β-strands. Detailed description and list of these tools are available in section Materials and Methods and Supplementary Table 1. Indeed, we were able to determine possible TM regions in some of the amyloidogenic proteins involved in neurodegenerative pathology. For others, we suspect that they can still form TM channels when in the oligomeric state. Further on, in the same set of sequences we looked for the motifs that represent signatures for the binding to gangliosides and cholesterol, GBM and CBM, respectively. Lipid rafts are rich in cholesterol and gangliosides (Figure 1) (Sezgin et al., 2017), and both are the sites where membrane interaction often begins; as seen in viruses (Wilson et al., 2004; Verdiá-Báguena et al., 2012) or in the direct pore formation through APs (Di Scala et al., 2016). The association of oligomeric α-synuclein with plasma membrane of hippocampal neurons was demonstrated to induce the formation of pore-like structures (Li et al., 2020). Furthermore, the results of Pacheco et al. (2015) go in line with the data of β-amyloid, another experimentally confirmed amyloidogenic pore forming peptide (Sepúlveda et al., 2014). Models of rather mobile Aβ channels have been proposed already in 2007 by the Nussinov group, who used molecular dynamics simulations (Jang et al., 2007, 2009, 2016; Capone et al., 2012). The simulations indicated that β-sheet channels might break into loosely associated mobile β-sheet subunits. The preferred channel sizes (16- to 24-mer) were compatible with electron microscopy/atomic force microscopy-derived dimensions (Jang et al., 2009).

**Figure 2:**
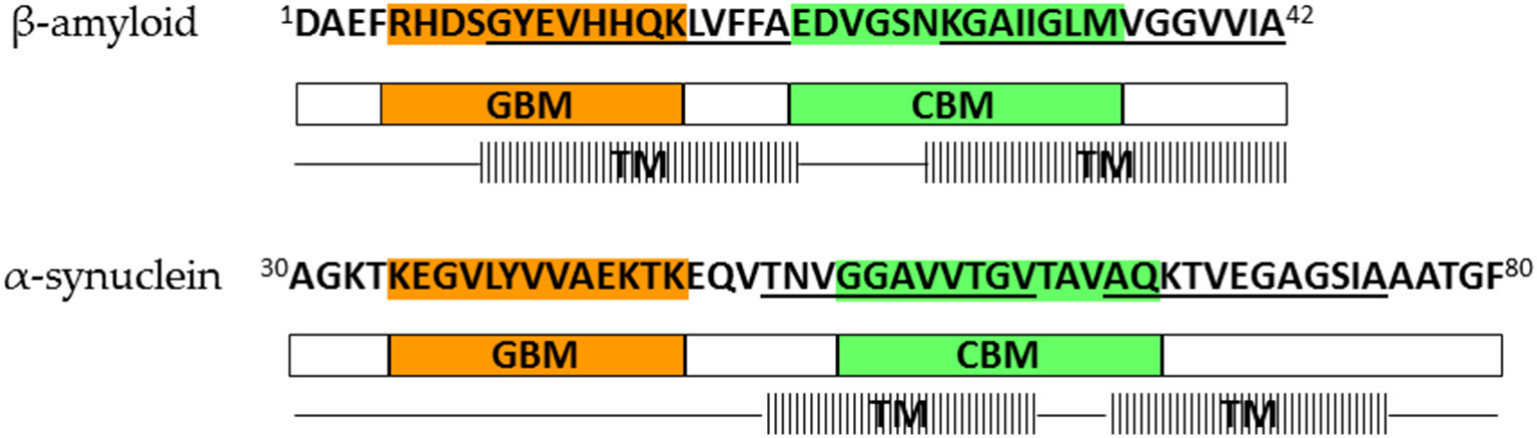
Lipid-binding domains and transmembrane regions in β-amyloid and α-synuclein that are involved in amyloid pore formation; orange—ganglioside-binding motif (GBM), green—cholesterol-binding motif (CBM), line pattern—transmembrane region (TM). Representation is based on results of Tsigelny et al. (2012), Di Scala et al. (2016), and Ciudad et al. (2020).

**Figure 3:**
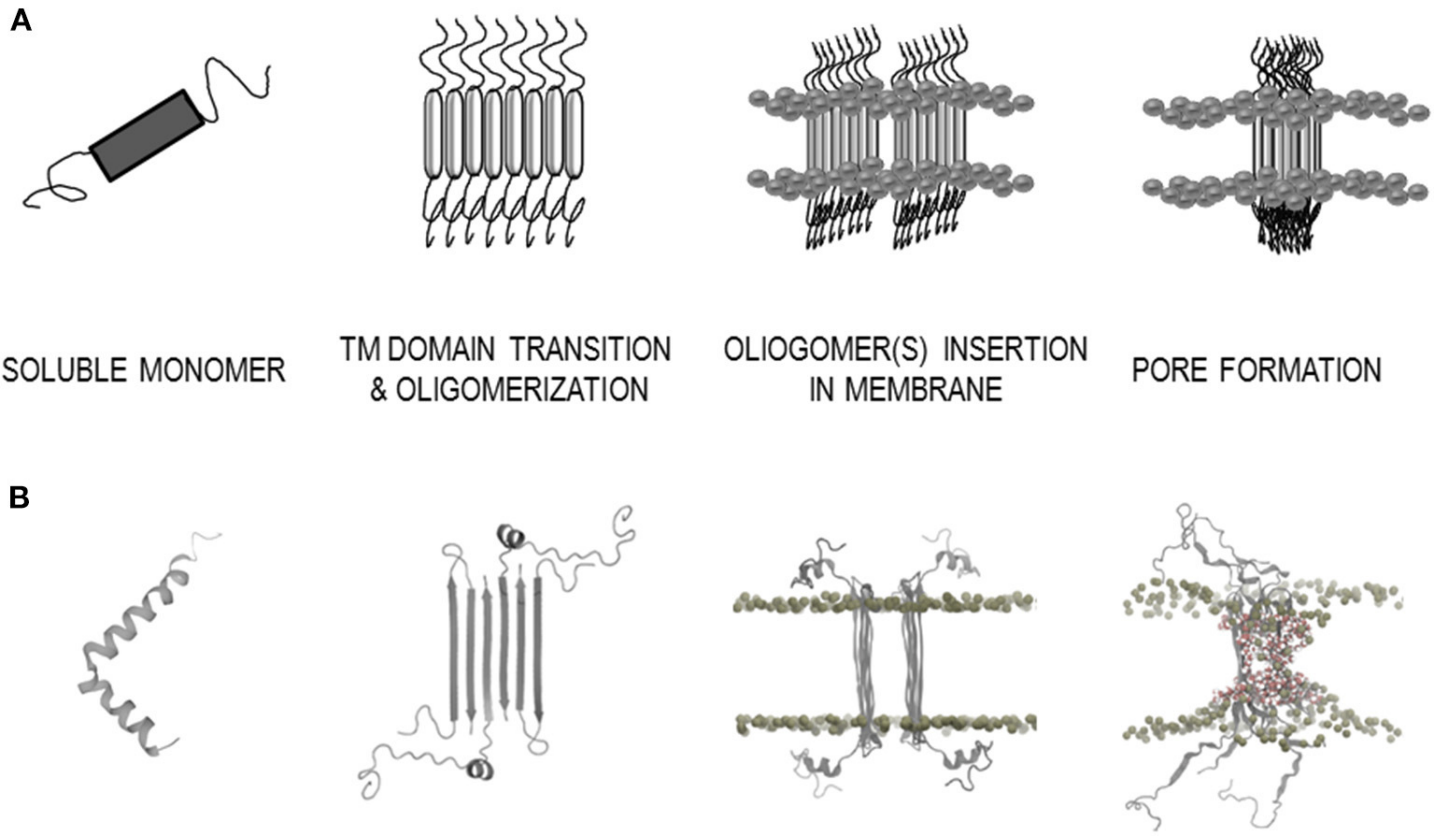
Model of the amyloid-membrane disruption mechanism based on a case study of β-amyloid (Aβ). **(A)** General scheme of membrane disruption; monomer secondary structure transition with aggregation into oligomer(s) and pore formation. **(B)** Structural data of membrane disruption by Aβ; soluble monomer α-helix (PDB: 1IYT), β-sheet structure of Aβ tetramer arrangement in membrane environment (PDB: 6RHY), MD simulations in DPPC membrane bilayer of Aβ octamer (Ciudad et al., 2020).

Further *in vitro* experiments have shown that AP formation involves both membrane lipids, ganglioside and cholesterol, that physically interact with amyloid proteins through specific structural motifs (GBM and CBM) (Jang et al., 2007, 2009, 2013; Di Scala et al., 2016; Dong et al., 2017). Mutation or deletion of these motifs abolished pore formation in α-synuclein (Parkinson's disease) and Aβ (Alzheimer's disease). Moreover, both peptides did no longer form Ca^2+^-permeable pores in the presence of drugs that target either cholesterol or ganglioside or both membrane lipids, indicating that gangliosides and cholesterol cooperate to favor the formation of AP through a common molecular mechanism (Di Scala et al., 2016). Figure 2 highlights the α-synuclein and β-amyloid domains that were confirmed by *in vitro* experiments to be involved in AP formation. Based on studies of how the β-amyloid tetramer and α-synuclein octamer insert into membranes (Tsigelny et al., 2012; Ciudad et al., 2020) we propose a possible common mechanism of membrane AP formation for other AFPs (Figure 3).

## Materials and Methods

The data set for this *in silico* experiment was generated by an extensive literature search for human proteins with a known amyloidogenic mode of action. A total of 30 proteins were selected for this study. The amino acid sequences of these proteins were compiled from UniProtKB database (https://www.uniprot.org/). The list of proteins and UniProtKB codes are shown in Table 1. The detailed protein descriptions and amino acid sequence representation and results of TM regions predictions, amyloidogenic regions, GBM and CBM regions are available in Supplementary Tables 1, 5).

### Prediction of Amyloidogenic Sequence Fragments and Propensity to Form Amyloid

Using the AmyPro database, we screened selected amino acid sequences against the entire collection of validated amyloidogenic sequence fragments to predict amyloidogenic regions within proteins (Varadi et al., 2018) (https://amypro.net). The database is publicly accessible and provides the boundaries of experimentally validated amyloidogenic sequence regions. Additional data are available, such as the functional relevance of the proteins and their amyloid state, experimental techniques used in the amyloid state studies, and relevant data transferred from the UniProt database.

Furthermore, the consensus method for the prediction of the amyloid propensityAmylPred2 (Tsolis et al., 2013) (http://aias.biol.uoa.gr/AMYLPRED2), was implemented in our data set. The FASTA format of the sequences was used as input. The consensus of different methods specifically developed for the prediction of features related to the formation of amyloid fibrils was generated for each protein. In this work a consensus of at least four methods was used.

RFAmyloid is a platform for protein sequence analysis based on machine learning approaches (Niu et al., 2018) (http://server.malab.cn/RFAmyloid). With the RFAmy classifier we estimated the propensity for amyloid based on the input of selected amino acid sequences in FASTA format. The predictions are based on the training set of original protein sequences from the Uniprot and AmyPro data sets and the technique of random forest for the classification of protein sequences (≤ 0.5 non-amyloid, >0.5 amyloid).

### Prediction of TM Regions

Several programs are available for the prediction of TM segments of proteins, either α-helices or β-strands. In this study different predictors were used, which are listed in Supplementary Table 2. All predictors are freely available online. PredαTM and PredβTM (Roy Choudhury and Novič, 2015) were developed in our laboratory and show reliable performance with reasonable predictions of α-helices or β-strands when compared to other predictors used. Reports of the benchmark analyzed are available in studies by Venko et al. (2017) and Roy Choudhury and Novič (2015). The PredαTM and PredβTM are two-layer predictors; the first layer is a classifier of TM segments, while the second layer is an adjustment of the border amino acids of the TM segments, based on the propensity of border amino acids in structurally solved TM proteins available in the PDB database (Roy Choudhury and Novič, 2009). The initial classifier for predicting α-helix TM segments was based on the artificial neural network algorithm (Pasquier et al., 1999), later both classifiers were upgraded by using the support vector machine algorithm (Venko et al., 2017). Algorithms PredαTM and PredβTM are using the sliding window approach (20 and 10 amino acids for α-helix and β-strand, respectively) and each segment is classified by the pre-developed SVM classifier as either transmembrane or non-transmembrane (Roy Choudhury and Novič, 2012).

By concept, α- or β-TM regions are segments of predominantly hydrophobic residues, which are energetically suitable for the hydrophobic membrane environment and have aromatic/charged residues at the membrane-water interface (terminal positions of the TM regions). In general, the identification of α- or β-TM regions can be approached by two different concepts: pattern-based or homology-based. By first, TM features are predicted based on algorithms using hydrophobicity scales or sequence similarity, by second, the prediction is based on algorithms that make a comparison with existing data from homologs. Therefore, in the first case the applicability for homologs and non-homologs is theoretically the same, while in the second case the probability of the prediction depends on the homology rate or is biased with it (Venko et al., 2017). Since all TM proteins with currently known high-resolution structures are strictly homomers and no mix assemblies of both TM segments have yet been determined, the predictors for each TM unit have been separated and developed separately to achieve a better precision in the predictions of the TM regions. Both types of predictors use different computational methods, which can generally be categorized into three classes: physico-chemical methods [PRED-TMR (Pasquier et al., 1999), BOMP (Berven et al., 2004)], statistical methods [TMpred (Hofmann and Stoffel, 1993)], and machine learning methods [HMMTOP (Tusnády and Simon, 2001), TMHMM (Krogh et al., 2001), MEMSAT-SVM (Nugent and Jones, 2009), PredαTM (Roy Choudhury and Novič, 2015), OCTOPUS (Viklund and Elofsson, 2008), B2TMPRED (Jacoboni et al., 2001), PRED-TMBB (Bagos et al., 2004), PredβTM (Roy Choudhury and Novič, 2015), TBBpred (Natt et al., 2004), BOCTOPUS2 (Hayat et al., 2016), PureseqTM (Wang et al., 2019), MPEx (Snider et al., 2009), ABTMpro (Cheng et al., 2005)]. In addition, it is proposed to apply a consensus approach for relevant predictions based on the analysis of the results of various currently available predictors. This type of consensus approach is already included in predictors such as CCTOP (Dobson et al., 2015), TOPCONS (Tsirigos et al., 2015) and ConBBPred (Bagos et al., 2005). Machine learning methods are regarding various performance analyses recognized as the most advanced and accurate (Roy Choudhury and Novič, 2015; Venko et al., 2017). Most often they are based on learning algorithms such as Support Vector Machines, Hidden Markov Models and Neural Networks. Interfacial hydropathy profile with White-Wimley scale was defined in MPEx (Snider et al., 2009). Further on, an FFPred3 (Cozzetto et al., 2016) server was used for feature-based function prediction and then a search for any membrane gene ontology domains was performed.

### Protein Sequence Screening for Cholesterol and Ganglioside Binding Motifs

All protein sequences were manually screened for the presence of CBM and GBM, as suggested by Fantini and colleagues (Fantini and Barrantes, 2013; Yahi and Fantini, 2014; Fantini et al., 2020). Cholesterol interacts with membrane lipids and proteins at the molecular/atomic scale, thus the consensus cholesterol binding motifs CRAC and/or CARC were characterized (Fantini and Barrantes, 2013). The CRAC domain is generally referred as Cholesterol Recognition/interaction Amino acid Consensus sequence present in the TM segment. This is motif of mandatory amino acid residues (L/V)-X1–5-(Y)-X1–5-(K/R). The CARC domain is similar to the CRAC sequence, but exhibits the opposite orientation (K/R)-X1–5-(Y/F)-X1–5-(L/V) from the N-term to the C-term (an inverted CRAC domain) (Fantini and Barrantes, 2013). Aside, a possible universal GBM is a variation of motifs consisting of a triad of mandatory amino acid residues such as (K/R)-Xn-(F/Y/W)-Xn-(K/R). While the Xn are intercalating segments of usually four to five residues, which can contain any amino acid, but often glycine (G), proline (P), and/or serine (S) residues (Yahi and Fantini, 2014).

## Results

### Propensity of 30 Proteins to Form Amyloid

Table 1 lists 30 human AFPs that we have selected for analysis based on experimental evidence that 29 of them form amyloid aggregates. We also included secretin as a putative negative control, since it was experimentally shown not to form amyloid, however, by predictive methods it proved to be highly amyloidogenic (Table 1, Supplementary Table 5). The functional category for each of the studied proteins is also shown in Table 1. Particularly, in addition to the 19 pathological AFPs, we also included three biologically non-relevant, seven functional amyloids and one negative control. However, for amyloidogenic protein cathelicidin the contrasting results among different sources were reported.

The chosen AFPs were examined for their propensity to form amyloid using the RFAmy predictor and for possible amyloidogenic sequence fragments using the AmyPro database and the AmylPred2 predictor. The results are shown in Table 1. A detailed graphical sequence representation is available in Supplementary Table 5, where the AmyPro amyloidogenic validated sequence fragments are marked. The RFAmy program classified 28 proteins as amyloids (probability >0.5). Using the AmyPRO database, the amyloidogenic sequence fragments were determined for 25 of the selected proteins; whereas for myoglobin and α-phosphatidyl inositol 3-kinase, homologous sequences from other species represented in the AmyPRO database were used. The only exceptions were stefin A, α-crystallin B chain, α-1-antichymotrypsin, cathelicidin, and secretin. For the above cases, it was crucial to use AmylPred2 (Tsolis et al., 2013) a consensus approach to predict amyloidogenic sequence fragments.

An interesting observation was made for α-1-antichymotrypsin since on one side, this protein was found in the amyloid plaques from the hippocampus of Alzheimer disease brains (Shoji et al., 1991; Padmanabhan et al., 2006; Tyagi et al., 2013) and known to promote Aβ deposition in plaques (Ma et al., 1994; Eriksson et al., 1995; Nilsson et al., 2001), thus confirming its pathological role. On the other side, we found a sequence homology with urocortin, a functional amyloid (Maji et al., 2009). Moreover, the antibacterial peptide cathelicidin was reported to act as immunomodulator that can contribute to the development of autoimmune diseases (Kahlenberg and Kaplan, 2013) and promote inflammation (Takahashi et al., 2018). On the other side, its exhibits a protective role as an inhibitor of amyloid self-assembly of Aβ (De Lorenzi et al., 2017) and islet amyloid polypeptide (IAPP) (Armiento et al., 2020).

### Probability of Forming Transmembrane Secondary Structures

Potential TM regions, either α-helices or β-strands have been predicted from amino acid sequences of the 305 proteins. Interestingly, for almost all AFPs at least one α- or β-TM region was determined. Table 2 shows the regions that can form TM α-helices or β-strands. The results of the predictions for each TM predictor are shown in Supplementary Table 3. Table 2 lists only α- or β-TM regions that meet the following criteria: they are predicted with at least two or more TM predictors, or the predicted TM region characterizes the same residues and secondary structures as in experimentally solved 3D structure of the soluble native form. In Supplementary Table 3 we have highlighted the regions which may form TM α-helices (gray color) and the regions which may form TM β-strands (yellow color).

**Table 2:** Transmembrane α-helix and β-strand predictions for the set of 30 amyloid-forming proteins; in italics are TM regions with CBM.

**ID**	**Protein name**	**TM regions**		**ATMBpro probability**	
		**α-helix**	**β-strand**	**TM protein**	**α-helix /β-strand TM protein**
1	β-amyloid	23–38^*^	10–21, *30–40*	**0.831**	0.808/0.023
2	α-synuclein	61–76^*^	*33–42, 55–66*, 72–82	**0.132**	0.096/0.036
3	Prion protein	90–109, 198–216^*^	/	**0.896**	0.883/0.013
4	Tau protein	/	13–21, *324–334*, 359–368, 371–380	0.044	0.040/0.004
5	β-2 microglobulin	/	50–56, *60–70*	**0.270**	0.242/0.029
6	Cystatin C	*95–112*	*41–49*, 59–66	**0.980**	0.964/0.016
**7**	Transthyretin	104–119^*^	*26–35, 65–81*, 88–97, *105–111*, 114–122	0.001	0.001/0.000
8	lysozyme C	20–35^*^	*11–21, 34–42*, 51–60	0.017	0.015/0.001
9	IAPP-amylin	13–28^*^	*7–16*	0.034	0.027/0.007
10	Calcitonin	/	*4–12*	0.015	0.012/0.003
11	Prolactin	/	*81–91, 95–104, 162–171*	**0.415**	0.413/0.002
12	Insulin	/	*31–39*	0.057	0.049/0.008
13	TDP-43	385–400^*^	25–34, 54–63, *67–76, 147–155, 225–233*, 264–274, 342–351	**0.377**	0.369/0.008
14	Superoxide dismutase 1	/	*42–48*	0.004	0.004/0.000
15	Stefin B (cystatin B)	/	*35–43, 49–58*, 65–72	0.004	0.003/0.002
16	α-crystallin B chain	/	*25–34, 75–83*, 114–123	0.010	0.010/0.000
17	α-1-antichymotrypsin	50–68^*^, 376–392^*^	31–37, 55–65, 73–83, *180–195, 217–230*, 240–249	**0.171**	0.029/0.000
18	Stefin A (cystatin A)	/	*36–46, 53–59, 65–73*	0.002	0.001/0.001
19	Myoglobin	/	9–18, 29–37, 65–77	0.001	0.001/0.000
20	α-phosphatidylinositol 3-kinase	/	*8–14*, 38–44, *74–83*	0.043	0.025/0.018
21	Cathelicidin	**/**	1–9, *19–26*	0.003	0.003/0.000
22	Secretin	/	*6*−1*3*, 19–27	**0.151**	0.137/0.014
23	Corticoliberin	**/**	9–16	0.078	0.067/0.011
24	GIP—gastric inhibitory polypeptide	/	*22–30*	0.022	0.021/0.014
25	Urocortin	6–21^*^	9–16	**0.186**	0.186/0.046
26	α-crystallin A chain	/	*86–93*	0.017	0.017/0.000
27	Obestatin	/	*6–11*	0.054	0.029/0.025
28	Glucagon	/	*/*	0.015	0.009/0.006
29	Defensin-6	/	*/*	0.006	0.006/0.000
30	β-endorphin	/	*14–20*	0.037	0.024/0.013

* *Pore-lining helix estimation by MEMSAT-SVM predictor. Bold values indicates core amino acids residues in motif*.

For the majority of proteins, β-TM secondary structures were more likely, but the ATMBpro predictor favored α-helices in all cases. Compared to other TM predictors, the ATMBpro tool is more restrictive in predicting whether a protein has the potential to be TM or not, since only three of 30 proteins have a high probability of being TM proteins (>0.5), seven have a medium probability (≥0.1 and ≤ 0.5) and the rest have a very low probability (<0.1). Interesting are also the predictions of the MEMSAT-SVM predictor, which for 17 proteins emphasizes the α-TM regions as pore-lining helices. In general, only one α-TM region is predicted for the majority of proteins, while β-TM regions are predicted more frequently, usually up to three or even more regions per protein (Table 2, Supplementary Tables 3, 5). Furthermore, the interfacial hydropaty profiles of all analyzed proteins are represented in Supplementary Table 4. Noteworthy, the results of FFPred3 search for membrane feature-based functions showed some membrane gene ontology domains in almost all analyzed proteins (Supplementary Table 6).

### Cholesterol and Ganglioside Binding Motifs

For almost all 30 analyzed proteins cholesterol and ganglioside binding motifs were detected. In Table 3 the sequences of CBM and GBM according to codes [(L or V)-(1–5 residues)-(Y)-(1–5 residues)-(K or R)], [(K or R)-(1–5 residues)-(Y or F)-(1–5 residues)-(L or V)] (Fantini and Barrantes, 2013), and [(K or R)-(4–6 residues)-(F or Y or W)-(4–6 residues)-(K or R)] (Yahi and Fantini, 2014; Fantini et al., 2020) are listed. Moreover, on a schematic representation of each protein sequence, the GBM and CBM motifs are highlighted in orange and green colors, respectively (Supplementary Table 5).

**Table 3:** Presence of potential cholesterol and ganglioside binding motifs.

**ID**	**Protein name**	**Cholesterol binding motif** **(L/V)-X**_****1−5****_**-(Y)-X**_****1−5****_**-(K/R)** **(K/R)-X**_****1−5****_**-(Y/F)-X**_****1−5****_**-(L/V)**		**Ganglioside binding motif** **(K/R)-Xn-(F/Y/W)-Xn-(K/R)**	
1	β-amyloid A	5–12 16–24 22–35	**R**NDSG**Y**E**V** **K**LVF**F**AED**V**^a^ EDVGSNKGAIIGLM^a^, ^b^^*^	5–16	**R**HDSG**Y**EVHHQ**K**
2	α-synuclein	34–41 53–65	**K**EGVL**Y**V**V**^b^ GGAVVTGVTAVAQ^a^, ^b^^*^	34–46	**K**EGVL**Y**VVAE**K**T**K**
3	Prion protein	129–139 183–196 191–203	**R**ENMHR**Y**PNQ**V** **R**ESQAY**Y**QRGSSM**V** **R**GSSMVL**F**SSPP**V**^a^	3–15 183–194	**R**PKPGG**W**NTGGS**R R**ESQA**Y**YQ**R**GSS
4	Tau protein	5–10 327–334 434–441	**R**QE**F**E**V** **L**T**F**RENA**K**^b^ **K**LD**F**KDR**V**	322–334	**K**ETHKLT**F**RENA**K**
5	β-2 microglobulin	19–27 58–65 75–82	**K**SN**F**LNCY**V** **K**DWS**FY**L**L**^b^ **K**DE**Y**ACR**V**	58–68	**K**DWSF**Y**LLYY**T**
6	Cystatin C	25–31 31–36 36–47 84–94	**R**ALD**F**A**V** **V**GE**Y**N**K** **K**ASNDM**Y**HSRA**L**^b^ **K**AFCS**F**QIYA**V**	23–46	**R**RALD**F**AVGEYN**K**
7	Transthyretin	30–35 71–80 103–110	**V**HV**F**R**K**^b^ **V**EIDTK**Y**YW**K**^b^ **R**R**Y**TIAA**L**^a^, ^b^	35–48	**K**AADDT**W**EPFASG**K**
8	Lysozyme C	1–8 14–25 31–41 119–130	**K**V**F**ERCE**L** **R**LGMDG**Y**RGIS**L**^a^, ^b^ **L**AKWESG**Y**NT**R**^a^, ^b^ **R**DVRQ**Y**VQGCG**V**	21–33 107–119	**R**GISLAN**W**MCLA**K** **R**AWVA**W**RN**R**CQN**R**
9	IAPP-amylin	11–17	**R**LAN**F**L**V**^a^, ^b^	1–11	**K**CNTA**F**CATQ**R**
10	Calcitonin	9–18	**L**GT**Y**TQDFN**K**^b^	18–28	**K**FHT**F**PQTAI**R**
11	Prolactin	16–23 77–84 88–98 164–172	**R**DL**F**DRA**V** **K**D**F**LS**L**I**V**^b^ **R**SWNEPL**Y**H**LV**^b^ **R**LSAY**Y**N**LL**^b^	88–102 164–177	**R**SWNEPL**Y**HLVTEV**R** **R**LSAY**Y**NLLHCL**R**
12	Insulin	33–43	**V**EAL**Y**LVCGE**R**^b^	43–50	**R**GFF**Y**TP**K**
13	TDP-43	74–82 145–150 151–159 208–216 226–231	**V**VN**Y**P**K**DN**K**^b^ **K**G**F**GF**V**^b^ **R**FTE**Y**ETQ**V**^b^ **R**EFSQ**Y**GD**V**^b^ **R**A**F**AF**V**^b^	151–160 208–219	**R**FTE**Y**ETQV**K** **R**EFSQ**Y**GDVMD**V**
14	Superoxide dismutase 1	14–23 42–47	**V**QGIIN**F**EQ**K** **L**HG**F**R**V**^b^	23–36	**K**ESNGPV**K**V**W**GSI**K**
15	Stefin B (cystatin B)	33–37 39–47 48–56 80–89	**K**K**F**P**V**^b^ **K**AVS**F**KSQ**V**^b^ **V**AGTN**Y**FI**K**^b^ **L**SN**Y**QTN**K**	30–39 78–91	**K**ENKK**F**PVF**K** **K**PLTLSN**Y**QTNKA**K**
16	α-crystallin B chain	22–32 44–50 72–79 82–89	**R**LFDQ**F**FGEH**L**^b^ **L**SPF**Y**L**R** **K**DR**F**SVN**L**^b^ **K**H**F**SPEE**L**^b^	11–22 69–82	**R**RPFFP**F**HSPS**R** **R**LEKDR**F**SVNLDV**K**
17	α-1-antichymotrypsin	156–166 183–193 226–231 295–303 367–377	**L**IND**Y**VKNGT**R** **V**LVN**Y**IFF**K**^b^ **L**TIP**Y**F**R**^b^ **R**D**Y**NLNDI**L** **R**TIVR**F**NRPF**L**	154–166	**K**KLIND**Y**VKNGT**R**
18	Stefin A (cystatin A)	30–38 38–47 48–56 58–67 68–73 81–89	**K**TNET**Y**K**L**^b^ **K**LEAVQ**Y**KTQ**V**^b^ **V**AGTN**Y**YI**K**^b^ **R**AGDNK**Y**MH**L**^b^ **K**V**F**KS**L**^b^ **V**LTG**Y**QVD**K**	30–44 58–71	**K**TNET**Y**G**K**LAVQ**K** **R**AGDNK**Y**MHL**K**VF**K**
19	Myoglobin	43–50 136–141 141–151	**K**FDK**F**KH**L** **L**EL**F**R**K** **K**DMASN**Y**KE**L**	43–51	**K**FDK**F**KHL**K**
20	α-phosphatidylinositol 3-kinase	13–20 68–76 76–81	**L**YD**Y**KKE**R**^b^ **R**GD**F**PGTY**V**^b^ **V**E**Y**IG**R**^b^	11–20 68–81	**R**ALYD**Y**KKE**R** **R**GDRPGT**Y**VEYIG**R**
21	Cathelicidin	14–23 25–34	**K**IGKE**F**KRI**V**^b^ **R**IKD**F**LRNL**V**^b^	14–25	**K**IGKE**F**KRIVQ**R**
22	Secretin	/		/	
23	Corticoliberin	/		/	
24	GIP—gastric inhibitory polypeptide	16–27	**K**IHQQD**F**VNWL**L**^b^	/	
25	Urocortin	34–40	**R**II**F**DS**V**	/	
26	α-crystallin A chain	12–22 50–57 79–86	**R**TLGPF**Y**PSR**L** **R**QSL**F**RT**V** **K**H**F**SPED**L**^b^	66–79 89–100 104–116	**R**SDRDK**F**VIFLDV**K K**VQDD**F**VEIHG**K** **R**QDDHG**Y**ISREFH**R**
27	Obestatin	/		/	
28	Glucagon	18–26	**R**AQD**F**VQW**L**	/	
29	Defensin-6	/		/	
30	β-endorphin	/		/	

a *In α-TM region*.

b *In β-TM region*.

* *Experimentally defined. Bold values indicates > 0.15*.

Further on, the representative TM regions which include a CBM are underlined in Table 2. Namely, all 19 pathological AFPs were determined to possess at least one TM region, which fulfilled the criteria of including all three regions (TM, CBM, and GBM), while on the contrary; the functional AFPs and negative control do not satisfy above mentioned criteria (Table 4). Among the biologically non-relevant proteins α-phosphatidylinositol 3-kinase and stefin A possess TM regions with the fulfilled criteria (TM, CBM, and GBM), while myoglobin does not show TM regions, which would fulfill TM-CBM-GBM criteria.

**Table 4:** Representation of 30 amyloid-forming proteins (AFPs) according to amyloid category and fulfilled criteria of including all three domains (transmembrane, cholesterol, and ganglioside binding regions).

**Amyloid category**	**No. of AFPs**	**No. of AFPs fulfilled TM-CBM-GBM criteria**
Pathological	19	19
Functional	7	0
Biologically non-relevant	3	2
Negative control	1	0

## Discussion

In accordance with the proposal of Dobson (2002) and Chiti and Dobson (2017) that any protein under proper conditions can transform into amyloid state, we determined the propensity to form amyloids for all 30 AFPs (Table 1). However, the kinetics of amyloid fibril formation is dictated by stability of the protein and its tendency to form folding intermediates (Dobson, 2017) as seen for example in the case of stefin B against stefin A (Jenko et al., 2004).

Similarly, it is believed that most if not all amyloid proteins can form oligomers, which exert toxicity via membrane binding and perforation (Bucciantini et al., 2002; Stefani and Dobson, 2003). The channel theory of Alzheimer's disease (AD) was proposed in 1993 by Arispe et al. (1993a,b), who stated that β-amyloid (Aβ) peptide perforates the plasma membrane, leading to the entry of Ca^2+^ ions and downstream signaling, which eventually causes cytotoxicity (Pacheco et al., 2015; Di Scala et al., 2016). Not long ago, the structure of the Aβ oligomer that could perforate the plasma membrane was proposed based on molecular dynamics and solid state NMR (Ciudad et al., 2020), which contributes to a better understanding of the possible mechanism of toxicity in AD (Press-Sandler and Miller, 2018). Meanwhile, Lashuel et al. (2002) and Lashuel and Lansbury (2006) describe that APs are formed by many amyloidogenic proteins and are a common source of amyloid-induced toxicity. The mechanism of their formation is still not well-understood and the imaging of pores in living cells remains an open issue. However, not so recent ago APs by Aβ were confirmed in living cells (Bode et al., 2017) and the membrane repair response was induced by Aβ in *C. elegans* model (Julien et al., 2018).

In order to get a deeper understanding of amyloid membrane interaction, we used different bioinformatics and machine learning tools to predict amyloidogenic (Table 1) and TM regions (Table 2, Supplementary Table 3) in a set of 30 selected proteins, all associated with protein misfolding and aggregation into amyloid fibrils (Sawaya et al., 2007). Since machine learning approaches are best suited to solve problems in the absence of general theories (i.e., large amounts of data with noisy patterns), they are ideal for usage in the case of protein complexity. According to the results of the α-TM region predictions the Memsat-SVM predictor is one of the most sensitive, since this predictor is the only one that predicts α-TM regions in 25 proteins. However, Memsat-SVM predictor in benchmark analyses in deed performed as one of the best TM predictors. In particular performs well at predicting the correct number of TM helices (95% accuracy) and also has a balanced number of over- and under predictions, which is favorable to avoid bias toward either type of prediction, and suggests good sensitivity while avoiding over predicting helices. By statistical parameters has very low rate of false positives (4%), for in comparison to others predictors, which have in general rate of false positives >10% (Nugent and Jones 2009; Venko et al., 2017). The TMpred and TMHMM predictors estimated α-TM regions in about one third of proteins, while the remaining α-TM predictors estimated α-TM regions in only three of the 30 AFPs. Such a difference in the sensitivity of α-TM regions predictors is somewhat surprising, since most predictors for α-TM regions in benchmark analysis showed very high (≥90%) sensitivities (Venko et al., 2017). Anyhow, since of the amphipathic nature of the β-TM regions, the hydrophobicity alone is an inefficient differentiating factor, so in advanced β-TM predictors the inclusion of non-linear statistics and evolutionary profiles was added to optimize predictions (Bagos et al., 2005). The recent benchmark analysis for β-TM predictors presented in Venko et al. (2017) shows that the PredβTM predictor based on machine-learning methodology currently outperforms all state-of-art β-TM region prediction methods. Indeed, in 27 proteins β-strand TM regions were predicted with the PredβTM predictor. Some predictors were less sensitive (PRED-TMBB, B2Tmpred, MEPx-BB), while the remaining predictors did not predict any β-TM regions. This fact is consisted with the estimated sensitivity of the separate β-TM predictors in the study by Roy Choudhury and Novič (2015). In general, the comparison between amyloidogenicity and TM potential is evident for 21 AFPs. As shown in Table 2, at least one or more TM regions in each protein were predicted by several TM region predictors. However, the estimation with the ABTMpro predictor shows that most of them have a very low TM probability score. It is interesting that those ones which appear in amyloid or neurodegenerative diseases (such as Aβ, cystatin C and prion) have a high probability of behaving as TM proteins (Di Scala et al., 2016; Kandel et al., 2017). However, in most AFPs sequences both TM secondary structures α-helices and β-strands were predicted, thus it is hard to decide, which one is the preferred one in AP formation. Tsigelny et al. (2012) in their study of α-synuclein membrane interaction provided reasonable explanation of this ambiguity and pointed out that during membrane binding and TM transition both secondary structures possibly occur. Their computational analysis of α-synuclein TM scores predicted that the region including residues 64–79 resembles a TM helix, since this region contains a significant number of hydrophobic residues that could play a critical role during the process of membrane penetration. Further analysis shows that α-synuclein α-helical conformer penetrates the membrane and undergoes change in the secondary structure with portions of the α-helices converting into π-helices and eventually extending into β-strands (Sepúlveda et al., 2014).

For the 42 amino acids long β-amyloid (Aβ) they have recently by using a combination of molecular dynamics calculations and solid state NMR measurements determined the structure of the pore-forming oligomers in lipid environment [tetramers/octamers, PDB: 6RHY (Ciudad et al., 2020)] (Figure 3B). Using several TM predictors, we showed that for the Aβ peptide, both types of TM regions are possible (Table 2, Supplementary Table 3). Although the ATMBpro predictor seems to prefer the α-helix structure, Ciudad et al. (2020) showed in their semi-empirical study that β-strand structures might be involved in the oligomerization and pore formation by Aβ. The two regions (G9-A21 and K28-A42) were confirmed as TM segments and both in formation of β-strands (Ciudad et al., 2020). Thus, predictors defined the second β-strand segment (A30-V40) correctly, while the first segment was determined by B2TMpred (Y10-A21) and partly with PRED-TMBB (F4-H14) predictor.

The architecture of the Aβ tetramer [PDB: 6RHY (Ciudad et al., 2020)], which could form pores in membranes, showed that the secondary structure in the oligomer differs from that present in the soluble monomers. Two α-helices were determined in the monomer [PDB: 1IYT (Crescenzi et al., 2002)], while antiparallel β-strands are present in the tetramer. This seems to be consistent with α to β secondary structure transition on the membrane. Indeed, it is known that many amyloidogenic proteins transform into β-sheet conformation before aggregating into amyloid fibrils. This type of oligomers with higher β-structure of Hyp was shown to be more toxic (Evangelisti et al., 2016). It is possible that α-helical parts on the lipid rafts, rich in gangliosides, undergo a secondary structure transition from α to β. It is remarkable that possible ganglioside binding sites can be detected for 25 analyzed AFPs (Table 3, Supplementary Table 5). For example, comparing human stefins B and A, such a site is found at the end of the α-helical part of stefin B (K30-K39), whereas in stefin A it prolongs up to residue 44 (K30-K44) (which is an overpredicted α-helix (Žerovnik et al., 1999). Both proteins also demonstrate another potential ganglioside binding site from residues K56-R68 (stefin B) and R58-K71 (stefin A), which resides in the third β-strand of native soluble form [PDB: 1DVD, (Žerovnik et al., 2011)]. The importance of cholesterol and ganglioside-binding domains in AP formation was experimentally shown in study of Di Scala et al. (2016). Mutation or deletion of these motifs in α-synuclein and Aβ abolished pore formation. Therefore, in our study another remarkable property of AFPs was observed, namely, that also the cholesterol binding domains in TM regions were found in 25 AFPs (Table 3). In general the CRAC and/or CARC domains were detected in TM regions, but occasionally some mispredicted unrealistic cholesterol binding domains outside TM regions were also observed. This is in accordance with observations of Fantini and Barrantes (2013).

The schematic mechanism of Aβ pore formation based on the possible tetramer structure (Ciudad et al., 2020) is depicted in Figure 3B. Derived from the case of Aβ we propose a more general mechanism (Figure 3A). This may apply to most amyloidogenic proteins, including cystatin C and the stefins A and B, which are involved in the typical amyloid disease; the hereditary amyloid angiopathy (cystatin C) or in a progressive myoclonal epileptic syndrome EPM1 with features of neurodegeneration (stefin B) and are non-physiological (such as stefin A) serving as model proteins in our previous work on protein aggregation to amyloid fibrils (stefins A and B) (Žerovnik et al., 1999, 2010; Anderluh and Žerovnik, 2012). In Parkinson's disease, the calcium-permeable pores formed by small oligomers of α-synuclein are thought the primary pathological species (Sepúlveda et al., 2014; Di Scala et al., 2016; Press-Sandler and Miller, 2018; Li et al., 2020). Our predictions for α-synuclein (residues 30–80) are in concordance with previously experimentally confirmed AP domains (Figure 2) (Sepúlveda et al., 2014). For the islet amyloid polypeptide and calcitonin experiments have also been conducted, which further confirm our assumption that many amyloidogenic proteins have potential to induce toxicity via pore formation (Press-Sandler and Miller, 2018). The islet amyloid polypeptide (IAPP or amylin) is a highly amyloidogenic peptide, and it has been hypothesized that transient membrane-bound α-helical structures of human IAPP are precursors of the amyloid deposits formation. The high-resolution structure of rat IAPP in the membrane-mimicking detergent micelles composed of dodecylphosphocholine was solved and α-TM region (A5-S23) was characterized (Nanga et al., 2009). The characterized regions are almost identical to our TM predicted regions; α-helix (A13-S28) or β-strand (C7-L16). While the MD simulations of possible structures of “amylin membrane channels” in various lipid bilayers using relatively large sizes of oligomers (12–36-mers) have been investigated and demonstrate the β-strands interfacing with the pore (Press-Sandler and Miller, 2018). The amphipathic α-helix was also experimentally determined in the membrane environment for the hormone calcitonin (T6-Y22) (Motta et al., 1991) and (S5-L19) (Hashimoto et al., 1999). The characterized regions are consistent with our TM predicted region for β-strand (L4-L12).

Furthermore, by using molecular dynamics and other computational methods the toxicity mechanism of transactive response DNA-binding protein 43 (TDP-43), which has the prion-like C-terminal domain (residues 258–414) and is believed to be a major component of neuronal inclusion bodies in amyotrophic lateral sclerosis, was studied. By the unbiased atomic-detailed molecular dynamics simulations, the C-terminal fragments of TDP-43 were observed to aggregate and form disordered-toroidal pores in a lipid bilayer (Chen et al., 2016). Apart, the interaction of tau protein with membranes was recently investigated experimentally, in aim to characterize the effect of the tau-membrane interactions on the function, aggregation, and toxicity of tau in neuronal cultures. Although, the atomic structures of tau oligomers are unknown and currently it seems that the lack of the structure might delay the future studies on tau oligomers on membrane surfaces (Press-Sandler and Miller, 2018). Interestingly, the only relevant region predicted in tau protein is β-TM region (324–334) and is located in exon 10, which contains the microtubule-binding region and is only expressed in 4-repeat (4R) tau isoforms, while 3-repeat (3R) tau isoforms are produced without exon 10 (Kametani and Hasegawa, 2018). The rest of the proteins that were analyzed in our study, up to our knowledge, do not have experimentally solved 3D structures of their oligomers in membrane environments.

It is worth to highlight a comparison of amyloidogenic peptides with the antimicrobial ones. Although they do not share common sequences, typical secondary structures, or the same biological activity, both exhibit membrane-disruption ability to induce cytotoxicity (Zhang et al., 2014). The interactions with membranes may be on the surface or within the cell membrane. Consequently, membrane interactions may affect the structure of the amyloid species and at the same time, the structure of the membrane that leads to cytotoxicity. Despite the existence of different membrane disruption mechanisms, the formation of TM pores appears to be a generic mechanism applicable to both antimicrobial and amyloidogenic membrane interacting peptides (Zhang et al., 2014; Press-Sandler and Miller, 2018). A comparison of different computationally modeled and experimental observed amyloid channels reveals several common features in channel structure and activity. Amyloid membranes channels preferably contain a U-shaped β-strand—turn-β-strand conformation (Zhang et al., 2014). In general, three models for the mechanism of membrane interaction/perforation by amyloid or antimicrobial peptides have been proposed: pore model, carpeting model, and detergent-like model (Zhang et al., 2014; Press-Sandler and Miller, 2018). However, the understanding of the molecular mechanisms of amyloidogenic proteins interaction with membranes remains a challenge to both experimental and computational studies.

## Conclusions

Even though all proteins may under certain conditions form amyloid state (according to Dobson, 2002), they differ in the propensity and likehood to form such a state, depending on thermodynamic and kinetic factors and environment, such as temperature, pH, reactive oxidative species—i.e., free radicals (ROS) and the crowding milieu. In our analysis we cannot predict all these factors but get by using various predictive methods a number expressing the propensity to transform into amyloid state (Table 1). From the functional point of view, among the 30 analyzed amyloid forming proteins (AFPs), we can differentiate those, which are a hallmark of disease and are termed “pathological” (19 cases), those that are biologically non-relevant (3), those that are “functional” (7) and a putative negative control (Table 1). The results of our study confirm a common feature of AFPs to possess regions of TM segments, either α-TM helices or β-TM strands, as proposed by several TM predictors (Table 2). Moreover, interactions of amyloidogenic proteins with membranes via lipid rafts rich in gangliosides and cholesterol are indicated (Table 3), as the predictions confirm such binding sites in all of the 19 pathological AFPs, while they are not fully present in functional amyloids (Table 4). Based on the membrane interaction and structural data of a generic oligomer type of an AFP (Aβ) leading to AP (Tsigelny et al., 2012; Ciudad et al., 2020), we suggest, that such mechanism of induced toxicity via AP formation could be indeed a generic property (Bucciantini et al., 2002). Since Ciudad et al. (2020) emphasize that toxicity arises from the hydrophilic residues located on the edges of the β-sheets, which lead to the formation of lipid-stabilized pores, the oligomerization and the α-TM helix or β-TM strand transition on the membrane surface (on lipid rafts) seem to be the common key events. Hopefully, in the near future stable TM regions that were defined in this study will be further confirmed experimentally for several amyloidogenic proteins. Thus, potentially, all AFPs can under certain circumstances form APs and become toxic. It depends, where and how this happens and if it leads to pathology or is transient, perhaps signaling proteotoxic stress to cells (Protter and Parker, 2016).

## Data Availability Statement

The original contributions presented in the study are included in the article/Supplementary Materials, further inquiries can be directed to the corresponding authors.

## Author Contributions

EŽ, VS, and MN: conceptualization. KV and VS: methodology and analysis. KV: software and visualization. KV, VS, and EŽ: investigation. KV and EŽ: writing—original draft preparation. MN and VS: writing—review and editing. VS: supervision. MN: project administration. MN and EŽ: funding acquisition. All authors have read and agreed to the published version of the manuscript.

## Conflict of Interest

The authors declare that the research was conducted in the absence of any commercial or financial relationships that could be construed as a potential conflict of interest.

## Abbreviations

AP: amyloid pore
APFs: amyloid-forming proteins
CBM: cholesterol binding motif
GBM: ganglioside binding motif
TM: transmembrane.
