# Supplementary material for: Prediction of Transmembrane Regions, Cholesterol, and Ganglioside Binding Sites in Amyloid-Forming Proteins Indicate Potential for Amyloid Pore Formation
Source: Front Mol Neurosci. 2021 Feb 10;14:619496. doi: 10.3389/fnmol.2021.619496 (PMC7902868; doi:10.3389/fnmol.2021.619496)
Supplement: Supplementary file 1 [file Data_Sheet_1.pdf]

Supplementary Material

Table S1. List of studied amyloid-forming proteins with their UniProtKB descriptions of cellular location, function, tissue specificity and related diseases.

Protein name	UniProtKB code	Cellular location	Function	Tissue specificity	Disease
**1**<br>β-amyloid	P05067 (A4_HUMAN)<br>672-713	Cell surface membrane, nucleus, endosome, extracellular region or secreted	Cell surface receptor and performs physiological functions on the surface of neurons relevant to neurite growth, neuronal adhesion and axonogenesis.	Highly expressed principally in brain in presynaptic terminals in the central nervous system.	Alzheimer disease, Hereditary localized amyloidosis, Cerebral amyloid angiopathy
**2**<br>α-synuclein	P37840 (SYUA_HUMAN)<br>1-126	Extracellular region or secreted, nucleus	Neuronal protein that plays several roles in synaptic activity such as regulation of synaptic vesicle trafficking and subsequent neurotransmitter release.	Expressed in the brain and in cerebrospinal fluid of adults. Expressed in all fetal tissues.	Parkinson disease, Dementia (Lewy bodies), Multiple system atrophy
**3**<br>prion protein	P04156 (PRIO_HUMAN)<br>23-253	Plasma membrane, Golgi apparatus	Primary physiological function is unclear. May play a role in neuronal development and synaptic plasticity or required for neuronal myelin sheath maintenance or promote myelin homeostasis through acting as an agonist for ADGRG6 receptor or play a role in iron uptake and iron homeostasis.	Found in high quantity in the brain of humans infected with neurodegenerative diseases.	Creutzfeldt-Jakob disease, Kuru, Fatal insomnia, Gerstmann-Straussler-Scheinker disease, Spongiform encephalopathy with neuropsychiatric features, Hereditary sensory and autonomic neuropathy
**4**<br>tau protein	P10636 (TAU_HUMAN)<br>1-441	Plasma membrane, cytosol, cytoskeleton	Promotes microtubule assembly and stability, and might be involved in the establishment and maintenance of neuronal polarity.	Expressed in neurons in the peripheral and central nervous system. Mostly found in the axons of neurons.	Alzheimer disease, Pick disease of the brain, Progressive supranuclear palsy, Frontotemporal dementia, Corticobasal degeneration
**5**<br>β-2 microglobulin	P61769 (B2MG_HUMAN)<br>21-119	Extracellular region or secreted	Component of the class I major histocompatibility complex (MHC). Involved in the presentation of peptide antigens to the immune system.	Low tissue specificity	Immunodeficiency, Hereditary visceral amyloidosis, Dialysis-related amyloidosis
**6**<br>cystatin C	P01034 (CYTC_HUMAN)<br>27-146	Extracellular region or secreted	Inhibitor of cysteine proteinases and is thought to serve an important physiological role as a local regulator of this enzyme activity.	Expressed in body fluids (saliva, cerebrospinal fluid, plasma), epididymis, vas deferens, brain, thymus,	Hereditary cerebral hemorrhage with amyloidosis, Age-related macular degeneration

				ovary.	
**7**<br>transthyretin	P02766 (TTHY_HUMAN)<br>21-147	Extracellular region or secreted	Thyroid hormone-binding protein that transports thyroxine from the bloodstream to the brain.	Detected in retina pigment epithelium, liver, serum and cerebrospinal fluid. Highly expressed in choroid plexus epithelial cells.	Senile systemic amyloidosis, Leptomeningeal amyloidosis, Carpal tunnel syndrome, Familial amyloid cardiomyopathy, Familial amyloidotic polyneuropathy
**8**<br>lysozyme C	P61626 (LYSC_HUMAN)<br>19-148	Extracellular region or secreted	Enzymatic activity of hydrolysis, transglycosylation and slight esterase activity. Bacteriolytic function in tissues and body fluids, where are associated with the monocyte-macrophage system and enhance the activity of immunoagents.	Expressed in salivary gland, blood.	Visceral amyloidosis
**9**<br>islet amyloid polypeptide	P10997 (IAPP_HUMAN)<br>34-70	Extracellular region or secreted	Selectively inhibits insulin-stimulated glucose utilization and glycogen deposition in muscle, while not affecting adipocyte glucose metabolism.	Expressed in pancreas. IAPP is found in pancreatic islets of type 2 diabetic patients and in insulinomas.	Type 2 diabetes, insulinoma
**10**<br>calcitonin	P01258 (CALC_HUMAN)<br>85-116	Extracellular region or secreted	Calcitonin causes a rapid but short-lived drop in the level of calcium and phosphate in blood by promoting the incorporation of those ions in the bones.	Expressed in dorsal root ganglion, parathyroid gland, thyroid gland.	Medullary carcinoma of the thyroid
**11**<br>prolactin	P01236 (PRL_HUMAN)<br>29-227	Extracellular region or secreted	Prolactin acts primarily on the mammary gland by promoting lactation.	Expressed in pituitary gland.	Pituitary prolactinoma
**12**<br>insulin	P01308 (INS_HUMAN)<br>25-54, 90-110	Extracellular region or secreted	Insulin decreases blood glucose concentration. It increases cell permeability to monosaccharides, amino acids and fatty acids. It accelerates glycolysis, the pentose phosphate cycle, and glycogen synthesis in liver.	Expressed in type B pancreatic cell.	Injection localized amyloidosis, Diabetes mellitus, Hyperproinsulinemia
**13**<br>TAR DNA-binding protein 43	Q13148 (TADBP_HUMAN)<br>1-414	Continuously travels in and out of the nucleus	RNA-binding protein that is involved in various steps of RNA biogenesis and processing. Preferentially binds, via its two RNA recognition motifs RRM1 and RRM2, to GU-repeats on RNA molecules predominantly localized within long introns and in the 3'UTR of mRNAs .	Expression is high in pancreas, placenta, lung, genital tract and spleen.	Amyotrophic lateral sclerosis, Frontotemporal lobar degeneration
**14**	P00441	Mitochondrion, Nucleus	Catalytic activity - destroys radicals within	Expressed in pons and liver.	Amyotrophic lateral sclerosis,

superoxide dismutase 1	(SODC_HUMAN) 2-154		the cells and which are toxic to biological systems. It has a tendency to form fibrillar aggregates in the absence of the intramolecular disulfide bond or of bound zinc ions. Aggregates may have cytotoxic effects.		Spastic tetraplegia and axial hypotonia
**15** stefin B (cystatin B)	P04080 (CYTB_HUMAN) 1-98	Nucleus	This is an intracellular thiol proteinase inhibitor. Tightly binding reversible inhibitor of cathepsins L, H and B.	Expressed in mouth mucosa and lymphoid tissue.	Myoclonic epilepsy
**16** α-crystallin B chain	P02511 (CRYAB_HUMAN) 1-175	Nucleus	May contribute to the transparency and refractive index of the lens. Has chaperone-like activity, preventing aggregation of various proteins under a wide range of stress conditions.	Expressed in myocardial tissue and detected in high concentrations in lens.	Cataract, Myofibrillar myopathy, Cardiomyopathy
**17** α-1- antichymotrypsin	P01011 (AACT_HUMAN) 24-423	Extracellular region or secreted	Physiological function is unclear, it can inhibit neutrophil cathepsin G and mast cell chymase, both of which can convert angiotensin-1 to the active angiotensin-2.	Synthesized in the liver and its concentration increases in plasma in the acute phase of inflammation or infection.	Found in the amyloid plaques from the hippocampus of Alzheimer disease brains.
**18** stefin A (cystatin A)	P01040 (CYTA_HUMAN) 1-98	Cytoplasm	An intracellular thiol proteinase inhibitor that has an important role in desmosome-mediated cell-cell adhesion in the lower levels of the epidermis.	Expressed in the skin throughout the epidermis and mouth mucosa.	Peeling skin syndrome (epidermolytic ichthyosis)
**19** myoglobin	P02144 (MYG_HUMAN) 1-154	Cytosol, Extracellular region or secreted	Serves as a reserve supply of oxygen and facilitates the movement of oxygen within muscles.	Expressed in heart muscle, skeletal muscle.	/
**20** phosphatidylinositol 3-kinase regulatory subunit alpha	P27986 (P85A_HUMAN) 1-84	Cytosol, Plasma Membrane, Nucleus, Golgi apparatus, Endoplasmic reticulum	Binds to activated (phosphorylated) protein-Tyr kinases and acts as an adapter, mediating the association of the p110 catalytic unit to the plasma membrane. Necessary for the insulin-stimulated increase in glucose uptake and glycogen synthesis in insulin-sensitive tissues.	Expressed in skeletal muscle and brain, and at lower levels in kidney and cardiac muscle.	Agammaglobulinemia, Immunodeficiency by impaired B-cell function and hypogammaglobulinemia
**21** cathelicidin	P49913 (CAMP_HUMAN) 132-170	Extracellular region or secreted	Binds to bacterial lipopolysaccharides (LPS), has antibacterial activity.	Expressed in bone marrow and testis and neutrophils.	/
**22**	P09683 (SECR_HUMAN)	Extracellular region or secreted	Hormone involved in regulation of the pH of the duodenal content, food intake and water	Expressed in intestine and blood. Serum secretin levels	/

secretin	28-54		homeostasis. Binding to secretin receptor (SCTR), a G-protein coupled receptor expressed in the basolateral domain of several cells. Acts as a key gastrointestinal hormone by regulating the pH of the duodenal content.	are increased after single-meal ingestion.	
**23**<br>corticoliberin	P06850 (CRF_HUMAN)<br>154-194	Extracellular region or secreted	Hormone regulating the release of corticotropin from pituitary gland. Induces NLRP6 in intestinal epithelial cells, hence may influence gut microbiota profile.	Produced by the hypothalamus and placenta.	Autosomal dominant nocturnal frontal lobe epilepsy
**24**<br>GIP- Gastric inhibitory polypeptide	P09681 (GIP_HUMAN)<br>52-93	Extracellular region or secreted	Potent stimulator of insulin secretion and relatively poor inhibitor of gastric acid secretion.	Expressed in duodenum.	/
**25**<br>urocortin	P55089 (UCN1_HUMAN)<br>83-122	Extracellular region or secreted	Acts in vitro to stimulate the secretion of adrenocorticotropic hormone (ACTH). Binds with high affinity to CRF receptor types 1, 2-alpha, and 2-beta. Plays a role in the establishment of normal hearing thresholds. Reduces food intake and regulates ghrelin levels in gastric body and plasma.	Keratinocytes in epidermis and the outer and inner root sheaths of hair follicles, epithelium of sebaceous and sweat glands, erector pili muscle, cutaneous blood vessel walls, cutaneous nerves and dermal mononuclear cells. Detected in plasma cells in the lamia propria in colon mucosa. Expressed in pituitary and adrenal glands.	/
**26**<br>α-crystallin<br>A chain	P02489 (CRYAA_HUMAN)<br>1-173	Nucleus	Contributes to the transparency and refractive index of the lens. In its oxidized form (absence of intramolecular disulfide bond), acts as a chaperone, preventing aggregation of various proteins under a wide range of stress conditions. Required for the correct formation of lens intermediate filaments as part of a complex composed of BFSP1, BFSP2 and CRYAA	Expressed in eye lens.	Cataract
**27**<br>obestatin	Q9UBU3 (GHRL_HUMAN)<br>76-98	Extracellular region or secreted	Obestatin is hormone that may have an appetite-reducing effect resulting in decreased food intake. May reduce gastric emptying activity and jejunal motility.	Highest expression level in stomach. All forms are found in serum as well.	/
**28**	P01275 (GLUC_HUMAN)	Extracellular region or secreted	Plays a key role in glucose metabolism and homeostasis. Regulates blood glucose by	Secreted in the A cells of the islets of Langerhans. Release	/

glucagon	53-81		increasing gluconeogenesis and decreasing glycolysis. A counterregulatory hormone of insulin, raises plasma glucose levels in response to insulin-induced hypoglycemia. Plays an important role in initiating and maintaining hyperglycemic conditions in diabetes.	is stimulated by hypoglycemia and inhibited by hyperglycemia, insulin, and somatostatin.	
**29** defensin-6	Q01524 (DEF6_HUMAN) 69-100	Extracellular region or secreted	Has very low antimicrobial activity against Gram-negative and Gram-positive bacteria. May protect cells against infection with HIV-1.	Expressed in Paneth cells of the small intestine.	/
**30** β-endorphin	P01189 (COLI_HUMAN) 237-267	Extracellular region or secreted	Hormone and endogenous orexigenic opiate.	Expressed in pituitary gland. Beta-endorphin is stored in separate granules in hypothalamic POMC neurons, suggesting that secretion may be under the control of different regulatory mechanisms.	/

Table S2. List of predictors of transmembrane regions.

Predictor	Description	Reference	Link
**PredαTM**	Predictor of alpha helical TM regions	Roy-Choudhury and Novič, 2015	http://transpred.ki.si
**CCTOP**	Constrained Consensus TOPology prediction server (α-helical TM protein, consensus of 10 methods - HMMTOP, Membrain, Memsat-SVM, Octopus, Philius, Phobius, Pro, Prodiv, Scampi, TMHMM)	Dobson et al., 2015	http://cctop.enzim.ttk.mta.hu
**HMMTOP**	predicting TM helices and topology of proteins	Tusnády and Simon, 2001	http://www.enzim.hu/hmmtop/html/document.html
**TMpred**	Prediction of TM Regions and Orientation	Hofmann and Stoffel, 1993	https://embnet.vital-it.ch/software/TMPRED_form.html
**TMHMM**	Prediction of TM helices in proteins	Krogh et al., 2001	http://www.cbs.dtu.dk/services/TMHMM-2.0
**PRED-TMR**	Prediction of TM regions in proteins	Pasquier et al., 1999	http://athina.biol.uoa.gr/PRED-TMR
**MEMSAT-SVM**	Membrane Helix Prediction	Nugent and Jones, 2009	http://bioinf.cs.ucl.ac.uk/psipred

**OCTOPUS**	Prediction of membrane protein topology and signal peptides by using combination of hidden Markov models and artificial neural networks	Viklund and Elofsson, 2008	https://octopus.cbr.su.se/
**TOPCONS**	Consensus prediction of membrane protein topology and signal peptides (OCTOPUS, Philius, PolyPhobius, SCAMPI, SPOCTOPUS)	Tsirigos et al., 2015	https://topcons.cbr.su.se/
**PredβTM**	Predictor of beta strand TM regions	Roy-Choudhury and Novič, 2015	http://transpred.ki.si
**PRED-TMBB**	Hidden Markov Model method for predicting and discriminating beta-barrel outer membrane proteins	Bagos et al., 2004	http://biophysics.biol.uoa.gr/PRED-TMBB
**ConBBPred**	Consensus Prediction of TM Beta-Barrel Proteins (PRED-TMBB, TM-BETA, B2TMpred, BETATM, HMM-B2TM, TBB-pred, ProfTMB)	Bagosh et al., 2005	http://bioinformatics.biol.uoa.gr/ConBBPRED
**B2TMpred**	Neural-Net-Based TM Beta-Barrel Predictions of Proteins	Jacoboni et al., 2001	http://gpcr.biocomp.unibo.it/cgi/predictors/outer/pred_outercgi.cgi
**BOCTOPUS2**	topology prediction of TM beta-barrel proteins	Hayat et al., 2016	http://boctopus.bioinfo.se
**TBB-pred**	TM Beta Barrel prediction server	Natt et al., 2004	http://crdd.osdd.net/raghava/tbbpred
**BOMP**	prediction of beta-barrel integral outer membrane proteins	Berven et al., 2004	http://services.cbu.uib.no/tools/bomp
**MEPx**	Membrane Protein Explorer	Snider et al., 2009	https://blanco.biomol.uci.edu/mpex
**PureseqTM**	TM topology prediction	Wang et al., 2019	http://pureseqtm.predmp.com
**ABTMpro**	prediction if a given protein sequence is TM protein (further predicton of probabilities of the protein being alpha helical TM protein or Beta Barrel TM protein)	Cheng et al., 2005	http://scratch.proteomics.ics.uci.edu

TM - transmembrane

Table S3. Transmembrane region predictions by several predictors (protein ID the same as in Table S1).

**TM predictor**	**TM region prediction for single protein**									
	**1**	**2**	**3**	**4**	**5**	**6**	**7**	**8**	**9**	**10**
**PredαTM**	18-37	0	100-110<br>195-216	0	0	0	0	0	0	0
**CCTOP**	0	0	o88-109i	0	0	0	0	0	0	0

			i194-214o							
**HMMTOP**	0	0	i91-112o<br>o194-216i	0	0	0	0	0	0	0
**TMpred**	o24-42i	i49-68o	o90-119i<br>i197-219o	0	0	i95-112o	i105-123o	i19-38o	i15-37	0
**TMHMM**	0	0	i88-108o<br>o198-216i	0	0	o95-112i	i105-123o	i16-38o	i13-30o	0
**PRED-TMR**	0	0	90-109<br>195-215	0	0	0	0	0	0	0
**OCTOPUS**	0	0	i196-216o	0	0	0	0	0	0	0
**TOPCONS**	0	0	o89-109i<br>i195-215o	0	0	0	0	0	0	0
**Memsat-SVM**	o23-38i*	i61-76o*	o196-217i*	i282-297o*	o21-36i*	o97-112i	o104-119i*	i20-35o*	i13-28o*	i6-21o
**PredβTM**	30-40	37-44<br>55-66<br>72-82	4-13	13-21<br>292-300<br>324-334<br>359-368<br>371-380	60-70	56-66	26-35<br>89-98<br>111-116	11-21<br>25-36	8-18, 26-35	4-13
**PRED-TMBB**	i4-14o,<br>o30-41i	o2-9i	o57-77i	i8-18o<br>o145-153i	i7-13o<br>o50-56i<br>i60-70o<br>o87-95i<br>(o95-99i)	i41-49o<br>(i56-66o)<br>o106-116i	i41-49o<br>o69-79i<br>i82-90o<br>o105-111i<br>i114-122o	i34-42o<br>o55-63i	0	i4-12o
score <2.965	3.005	2.944	3.096	2.967	3.008	2.947	2.949	3.141	3.111	3.08
**ConBBPred**	0	0	0	0	0	0	0	0	0	0
**B2TMpred**	10 -21<br>31-40	1-10<br>13-22<br>33- 42<br>44-53<br>55-67<br>76-85	87-96<br>98-112<br>124-135<br>162-171<br>182-197<br>205-219<br>221-230	25-34<br>119-130<br>322-333<br>359-368<br>370-379<br>428-437	47-56<br>58-70	23 -35<br>42 -57<br>59 -68	12-21<br>28-37<br>65-81<br>88-97<br>102-111<br>113-122	8-24<br>32-41<br>51-60<br>104-113	7-16<br>18-27	5-14<br>16-25
**BOCTOPUS2**	0	0	0	0	0	0	0	0	na	na
**TBBpred**	0	0	0	0	0	0	0	0	0	0
**BOMP**	0	0	0	0	0	0	0	0	0	0
**MPEx-BB**	0	56-57	0	0	58-69	60-65	73-78	37-43	0	0

							110-120			
**MPEx-TM**	0	0	197-215	0	1-72	0	105-123	0	11-23	0
**MPEx-hydropathy**	0	0	4-22<br>55-99<br>123-141<br>218-236	0	74-92	1-19<br>121-139	77-95<br>105-123	16-45<br>54-72	12-30	4-22
**PURESEQTM**	30-49	0	89-105<br>197-216	0	0	0	0	0	0	0
**ABTMpro**	helix	no TM	helix	no TM	no TM	helix	no TM	no TM	no TM	no TM
Probabilities for TM protein	0.831	0.132	0.896	0.044	0.270	0.980	0.001	0.017	0.034	0.015
Probabilities for TM helix	0.808	0.096	0.883	0.040	0.242	0.964	0.001	0.015	0.027	0.012
Probabilities for TM β strand	0.023	0.036	0.013	0.004	0.029	0.016	0.000	0.001	0.007	0.003

*pore-lining helix, o - outside cell, i - inside cell

**TM predictor**	**TM region prediction for single protein**							
	**11**	**12**	**13**	**14**	**15**	**16**	**17**	**20**
**PredαTM**	0	0	0	0	0	0	50-59	0
**CCTOP**	0	0	0	0	0	0	0	0
**HMMTOP**	0	0	0	0	0	0	i50-68o	0
**TMpred**	0	0	o23-41i<br>i56-75o<br>o312-336i<br>i385-404o	0	0	0	i50-69o<br>i376-392o	0
**TMHMM**	0	0	i380-400i	0	0	0	i50-69o<br>o375-390i	0
**PRED-TMR**	0	0	0	0	0	0	50-68	0
**OCTOPUS**	0	0	0	0	0	0	0	0
**TOPCONS**	0	0	0	0	0	0	0	0
**Memsat-SVM**	0	o31-46i	i227-242o*	o105-120i*	i39-54o*	i20-35o	i52-67o*<br>o377-392i*	i27-42o*
**PredβTM**	81-91<br>95-104	11-22<br>30-42	25-34<br>36-45	33-43<br>142-150	47-59	63-72<br>74-84	27-37<br>56-65	7-17<br>73-83

	165-174		54-63<br>68-79<br>125-135<br>147-159<br>225-236				72-82<br>135-145<br>180-190<br>238-247<br>280-292<br>305-314<br>319-331	
**PRED-TMBB**	i23-31o<br>o81-91i	i31-39o	i71-77o<br>o90-100i<br>i103-113o<br>o131-141i<br>i147-155o<br>o225-233i<br>(267-275)	i14-20o<br>(o29-37i)<br>(i42-48o)<br>o81-87i	i35-43o<br>o53-59i<br>i65-73o<br>o95-98	i2-9o<br>o27-37i<br>i40-48o<br>o75-83i<br>i89-97o<br>o112-124	i33-43o<br>o55-63i<br>i74-84o<br>o108-114i<br>i120-130o<br>o167-175i<br>o208-218i<br>i224-230o<br>o245-255i<br>(i366-372o)<br>i388-394o	i8-14o<br>o26-34i<br>i38-44o<br>(o78-85i)
score <2.965	3.047	3.281	2.99	2.98	3.011	3.061	2.988	2.957
**ConBBPred**	0	0	0	0	0	0	0	0
**B2TMpred**	16-26<br>28-37<br>39-51<br>76-91<br>95-104<br>115-124<br>126-135<br>162-171<br>179-188	27-36<br>38-47	1-10<br>25-34<br>52-61<br>67-76<br>101-110<br>118-127<br>144-153<br>189-198<br>205-214<br>225-234<br>251-260<br>264-274<br>342-351<br>372-381<br>386-395<br>397-408	41-50<br>113-122	31-47<br>49-58<br>63-72<br>81-97	25-34<br>74-83<br>90-105<br>114-123	21-30<br>32-43<br>57-67<br>73-84<br>92-103<br>110-119<br>121-130<br>134-144<br>156-165<br>180-195<br>217-230<br>238-254<br>262-278<br>292-301<br>303-312<br>329-342<br>351-360<br>365-374<br>386-395	3-12<br>14-23<br>32-44<br>74-83
**BOCTOPUS2**	0	0	0	0	0	0	0	0

**TBBpred**	0	0	0	0	0	0	0	0
**BOMP**	0	0	0	0	0	0	0	0
**MPEx-BB**	0	0	304-309<br>327-333<br>343-352	0	46-56	77-80<br>136	31-37<br>244-249	13
**MPEx-TM**	80-98	10-28	216-234<br>386-404	0	0	3-32<br>37-65	50-68<br>180-198<br>214-232<br>372-390	0
**MPEx-hydropathy**	5-23<br>108-126	6-24<br>31-49	25-43<br>59-77<br>216-234	0	37-55	9-27<br>43-61	24-42<br>50-68<br>180-198<br>288-306<br>372-390	26-44
**PURESEQTM**	0	0	0	0	0	0	0	0
**ABTMpro**	no TM	no TM	no TM	no TM	no TM	no TM	no TM	no TM
TM protein	0.415	0.057	0.377	0.004	0.004	0.010	0.171	0.043
TM helix	0.413	0.049	0.369	0.004	0.003	0.010	0.029	0.025
TM β strand	0.002	0.008	0.008	0.000	0.002	0.000	0.000	0.018

**TM predictor**	**TM region prediction for single protein**									
	**21**	**22**	**23**	**24**	**25**	**26**	**27**	**28**	**29**	**30**
**PredαTM**	0	0	0	0	0	0	0	0	0	0
**CCTOP**	0	0	0	0	0	0	0	0	0	0
**HMMTOP**	0	0	0	0	0	0	0	0	0	0
**TMpred**	0	0	0	0	0	i128-146o	0	0	0	0
**TMHMM**	0	0	0	0	0	0	0	0	0	0
**PRED-TMR**	0	0	0	0	0	0	0	0	0	0
**OCTOPUS**	0	0	0	0	0	0	0	0	0	0
**TOPCONS**	0	0	0	0	0	0	0	0	0	0
**Memsat-SVM**	0	0	0	0	o6-21i*	i21-36o	i5-20o*	i11-26o*	i13-28o*	i12-27o
**PredβTM**	0	8-16<br>20-32	9-16	22-30	9-16	25-34<br>70-77<br>86-93	3-15	0	20-29	0

**PRED-TMBB**	o1-9i<br>i15-29o	I1-7o<br>o20-16i	0	o25-33i	o5-13i<br>i16-24o	0	i5-13o	i6-14o	i11-17o	i15-23o
score <2.965	3.044	2.986	2.938	3.042	2.883	3.027	2.864	3.155	3.603	3.073
**ConBBPred**	0	0	0	0	0	0	0	0	0	0
**B2TMpred**	1-14<br>19-26	7-14<br>16-32	9-25<br>27-38	2-7<br>16-31<br>40-54	7-21<br>23-28<br>30-35	41-50<br>63-73<br>86-95<br>102-107<br>111-117<br>126-135	6-11<br>18-28	9-22<br>24-35	0	14-20<br>28-34
**BOCTOPUS2**	0	0	0	0	0	0	0	0	0	0
**TBBpred**	0	0	0	0	0	0	0	0	0	0
**BOMP**	0	0	0	0	0	0	0	0	0	0
**MPEx-BB**	0	0	9-10	0	7-10	0	6-11	0	0	0
**MPEx-TM**	0	0	0	0	0	0	0	0	0	0
**MPEx-hydropathy**	0	0	0	0	0	0	0	0	14-32	0
**PURESEQTM**	0	0	0	0	0	0	0	0	0	0
**ABTMpro**	no TM	no TM	no TM	no TM	no TM	no TM	no TM	no TM	no TM	no TM
TM protein	0.003	0.151	0.078	0.022	0.232	0.017	0.054	0.015	0.006	0.037
TM helix	0.003	0.137	0.067	0.021	0.186	0.017	0.029	0.009	0.006	0.024
TM β strand	0.000	0.014	0.011	0.001	0.046	0.000	0.025	/0.006	0.000	0.013

Table S4. Interfacial hydropathy profiles by White-Wimley scale

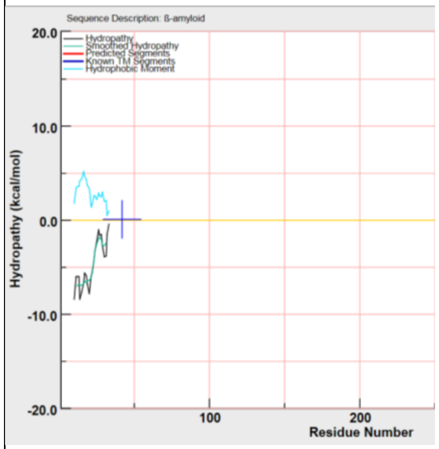

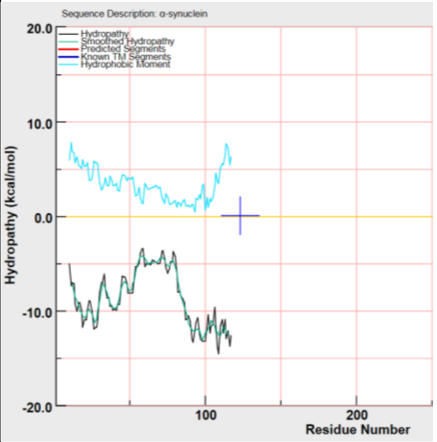

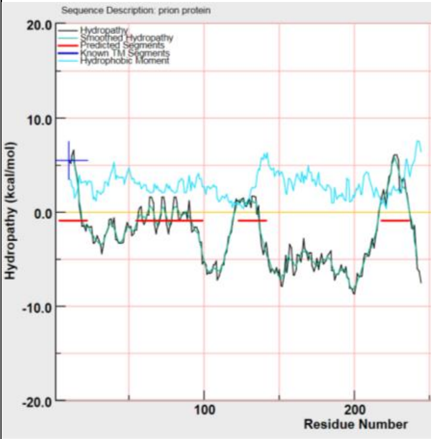

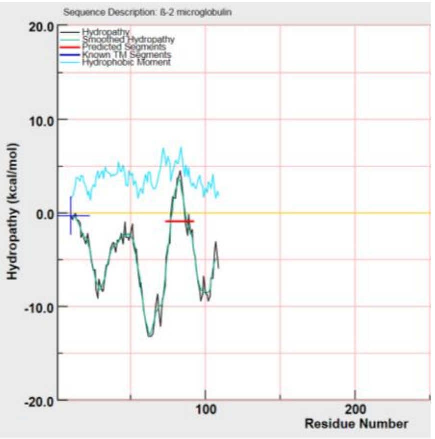

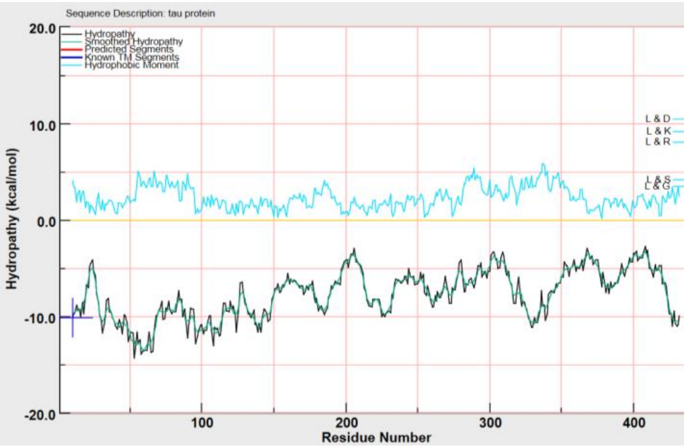

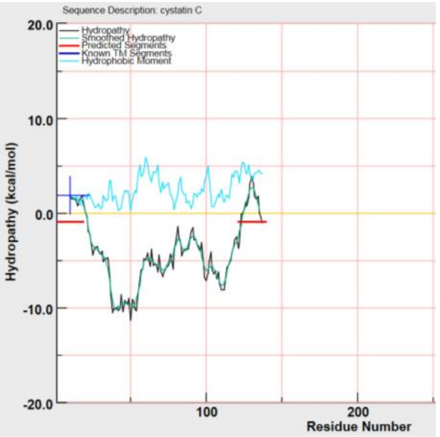

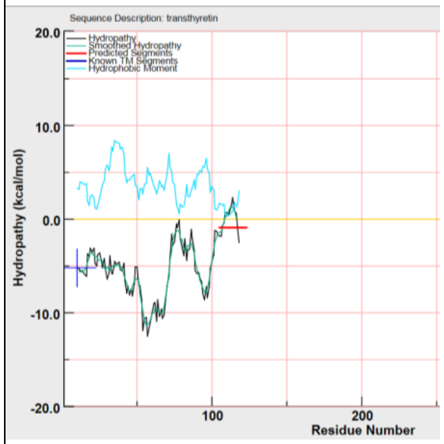

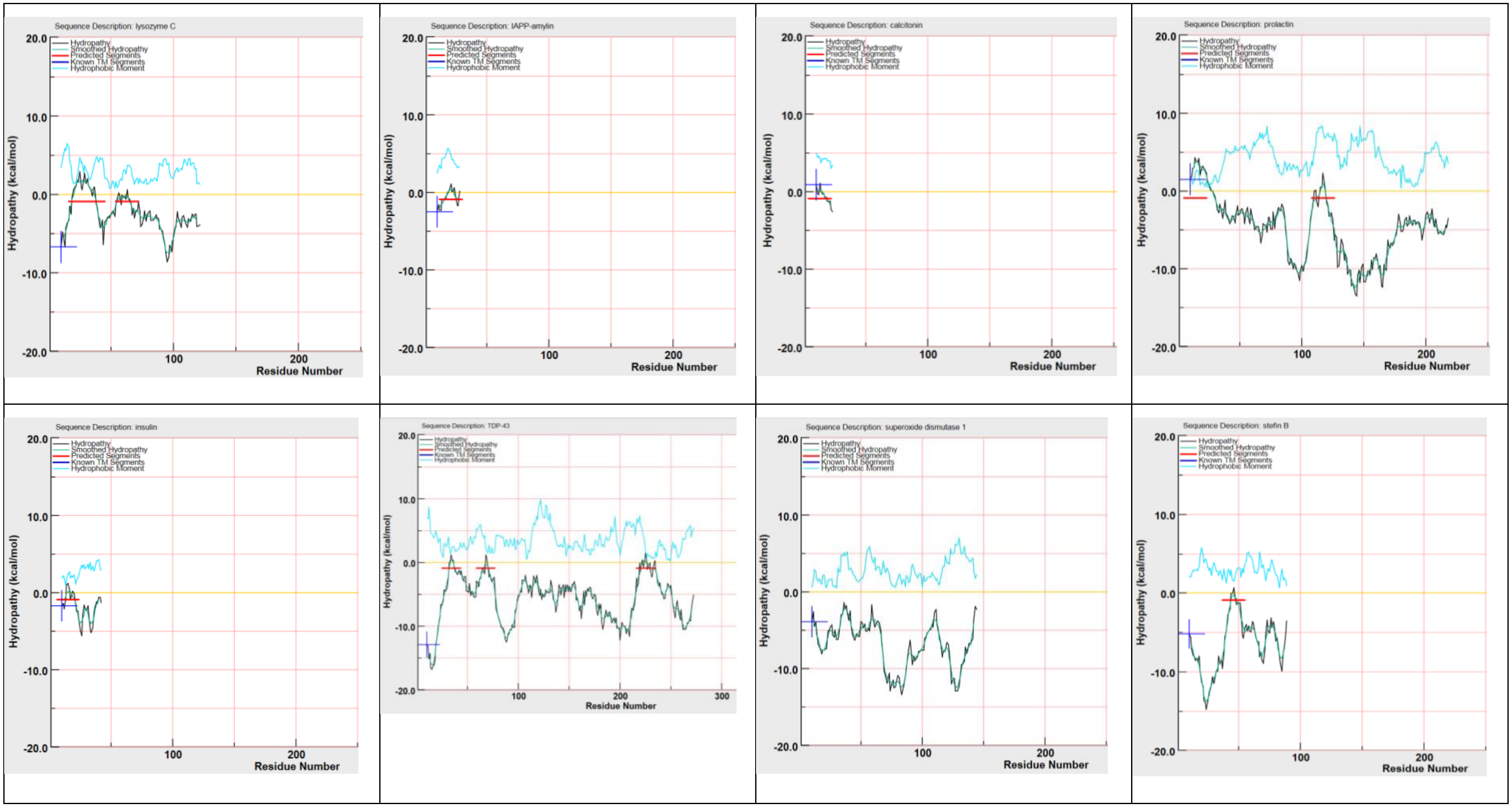
Sequence Description: lysozyme C
Sequence Description: IAPP-amylin
Sequence Description: calcitonin
Sequence Description: prolactin
Sequence Description: insulin
Sequence Description: TDP-43
Sequence Description: superoxide dismutase 1
Sequence Description: stefin B
Hydropathy
Smoothed Hydropathy
Predicted Segments
Known TM Segments
Hydrophobic Moment
Hydropathy (kcal/mol)
Residue Number
20.0
10.0
0.0
-10.0
-20.0
100
200
300

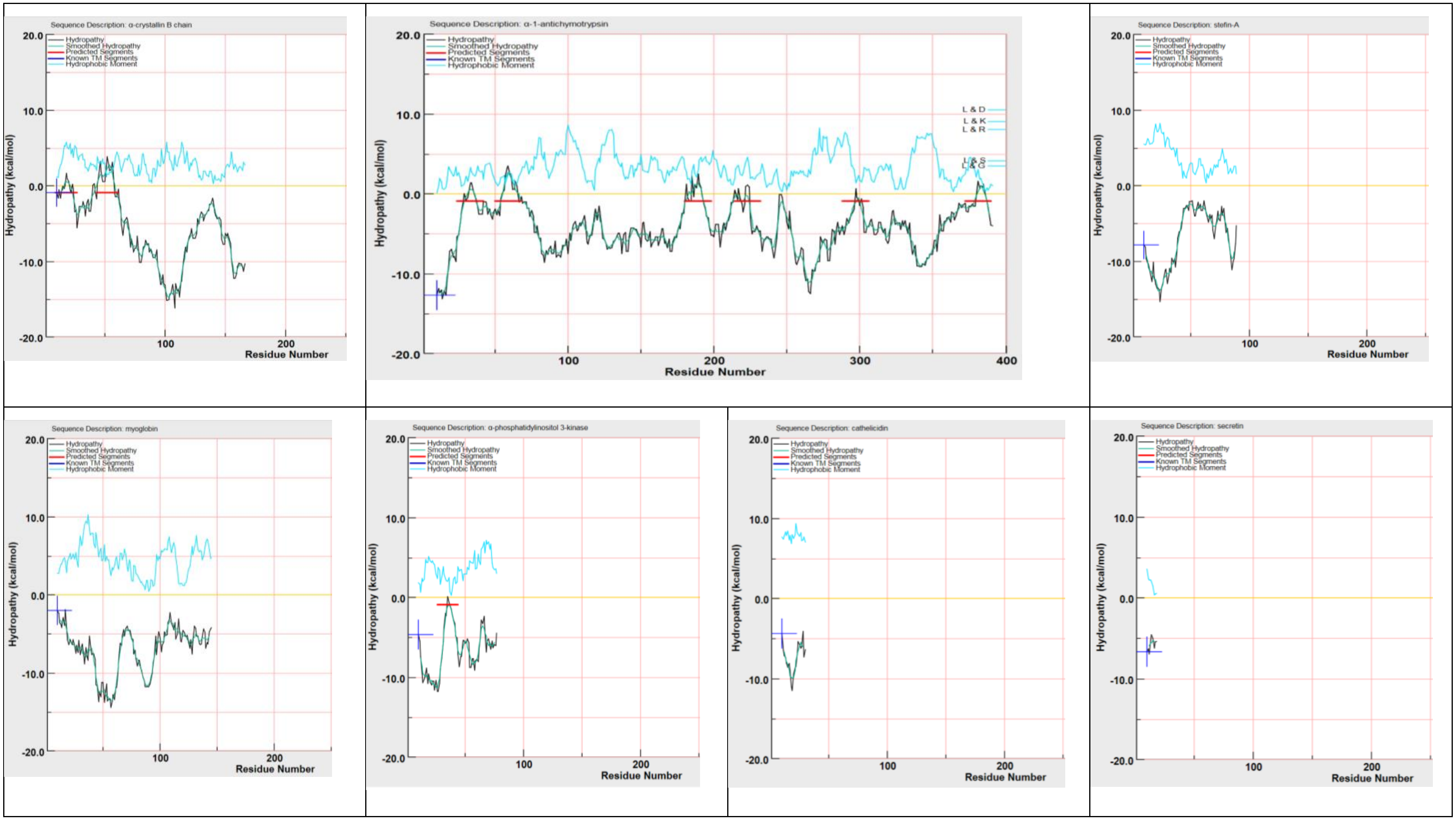
Sequence Description: α-crystallin B chain
Sequence Description: α-1-antichymotrypsin
Sequence Description: stefin-A
Sequence Description: myoglobin
Sequence Description: α-phosphatidylinositol 3-kinase
Sequence Description: cathelicidin
Sequence Description: secretin
Hydropathy
Smoothed Hydropathy
Predicted Segments
Known TM Segments
Hydrophobic Moment
Hydropathy (kcal/mol)
Residue Number
L & D
L & K
L & R
L & S
L & G

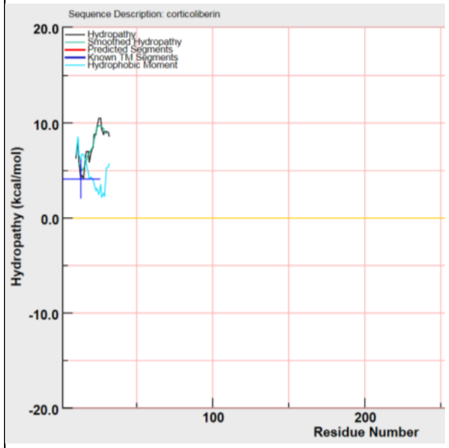
Sequence Description: corticoliberin
Hydropathy
Smoothed Hydropathy
Predicted Segments
Known TM Segments
Hydrophobic Moment
Hydropathy (kcal/mol)
Residue Number

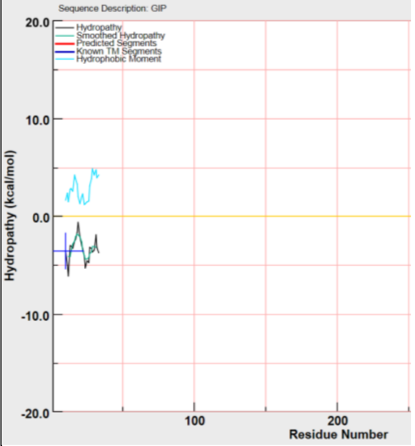
Sequence Description: GIP
Hydropathy
Smoothed Hydropathy
Predicted Segments
Known TM Segments
Hydrophobic Moment
Hydropathy (kcal/mol)
Residue Number

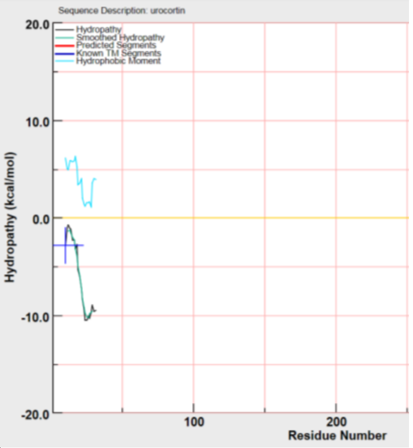
Sequence Description: urocortin
Hydropathy
Smoothed Hydropathy
Predicted Segments
Known TM Segments
Hydrophobic Moment
Hydropathy (kcal/mol)
Residue Number

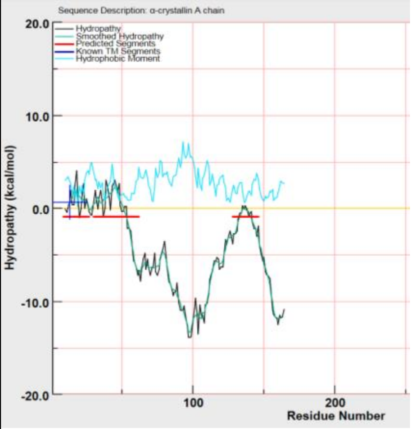
Sequence Description: α-crystallin A chain
Hydropathy
Smoothed Hydropathy
Predicted Segments
Known TM Segments
Hydrophobic Moment
Hydropathy (kcal/mol)
Residue Number

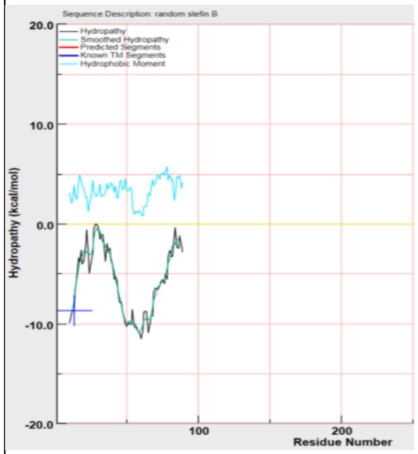
Sequence Description: random stefin B
Hydropathy
Smoothed Hydropathy
Predicted Segments
Known TM Segments
Hydrophobic Moment
Hydropathy (kcal/mol)
Residue Number

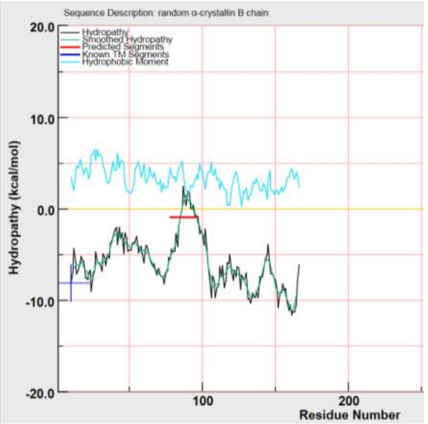
Sequence Description: random α-crystallin B chain
Hydropathy
Smoothed Hydropathy
Predicted Segments
Known TM Segments
Hydrophobic Moment
Hydropathy (kcal/mol)
Residue Number

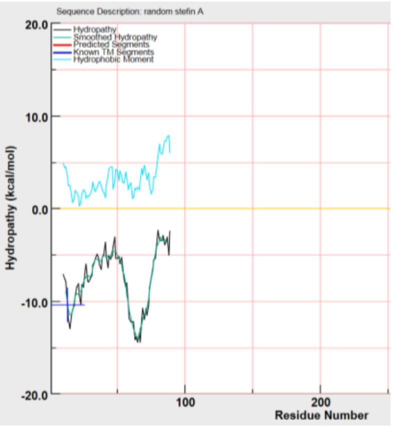
Sequence Description: random stefin A
Hydropathy
Smoothed Hydropathy
Predicted Segments
Known TM Segments
Hydrophobic Moment
Hydropathy (kcal/mol)
Residue Number

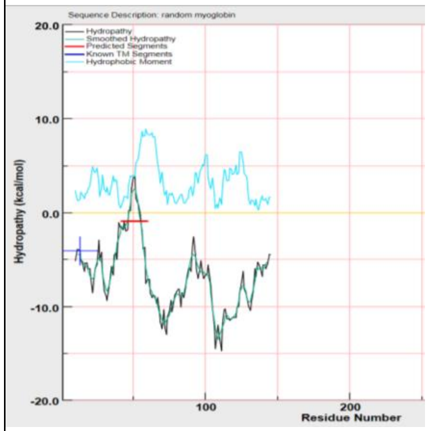
Sequence Description: random myoglobin
Hydropathy
Smoothed Hydropathy
Predicted Segments
Known TM Segments
Hydrophobic Moment
Hydropathy (kcal/mol)
Residue Number

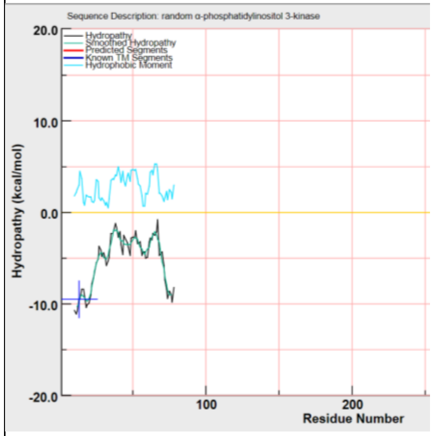
Sequence Description: random α-phosphatidylinositol 3-kinase
Hydropathy
Smoothed Hydropathy
Predicted Segments
Known TM Segments
Hydrophobic Moment
Hydropathy (kcal/mol)
20.0
10.0
0.0
-10.0
-20.0
100
200
Residue Number

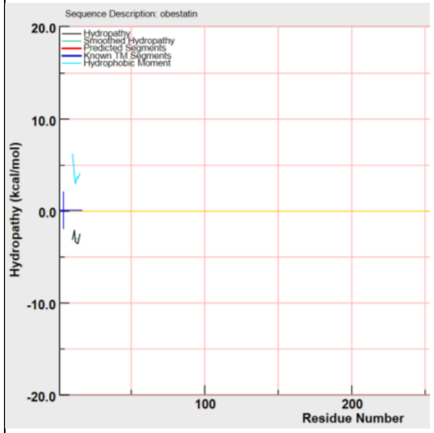
Sequence Description: obestatin
Hydropathy
Smoothed Hydropathy
Predicted Segments
Known TM Segments
Hydrophobic Moment
Hydropathy (kcal/mol)
20.0
10.0
0.0
-10.0
-20.0
100
200
Residue Number

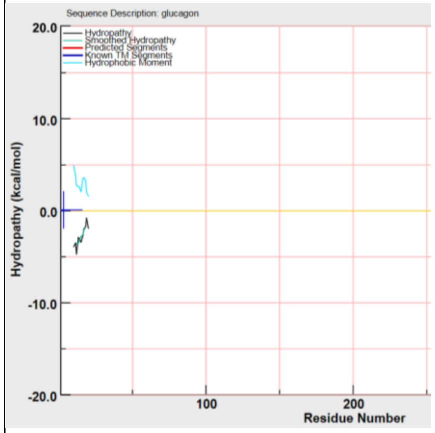
Sequence Description: glucagon
Hydropathy
Smoothed Hydropathy
Predicted Segments
Known TM Segments
Hydrophobic Moment
Hydropathy (kcal/mol)
20.0
10.0
0.0
-10.0
-20.0
100
200
Residue Number

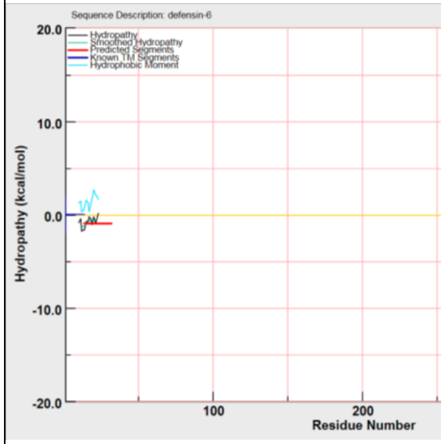
Sequence Description: defensin-6
Hydropathy
Smoothed Hydropathy
Predicted Segments
Known TM Segments
Hydrophobic Moment
Hydropathy (kcal/mol)
20.0
10.0
0.0
-10.0
-20.0
100
200
Residue Number

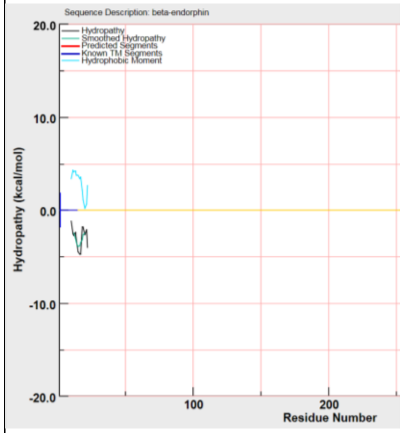
Sequence Description: beta-endorphin
Hydropathy
Smoothed Hydropathy
Predicted Segments
Known TM Segments
Hydrophobic Moment
Hydropathy (kcal/mol)
20.0
10.0
0.0
-10.0
-20.0
100
200
Residue Number

Table S5. Amino acid sequence representation of 30 amyloid-forming proteins and results of TM regions predictions, amyloidogenic regions, GBM and CBM regions.

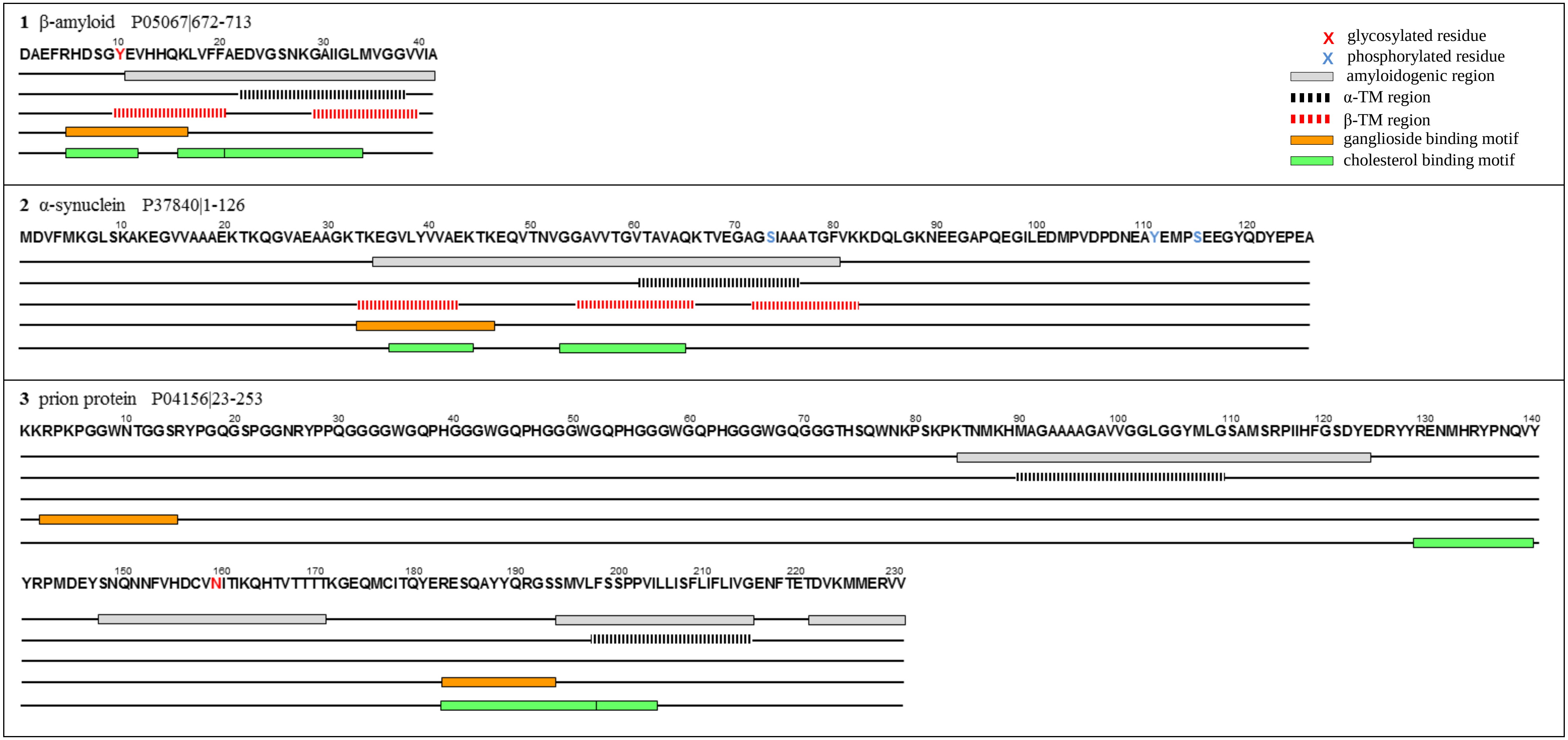

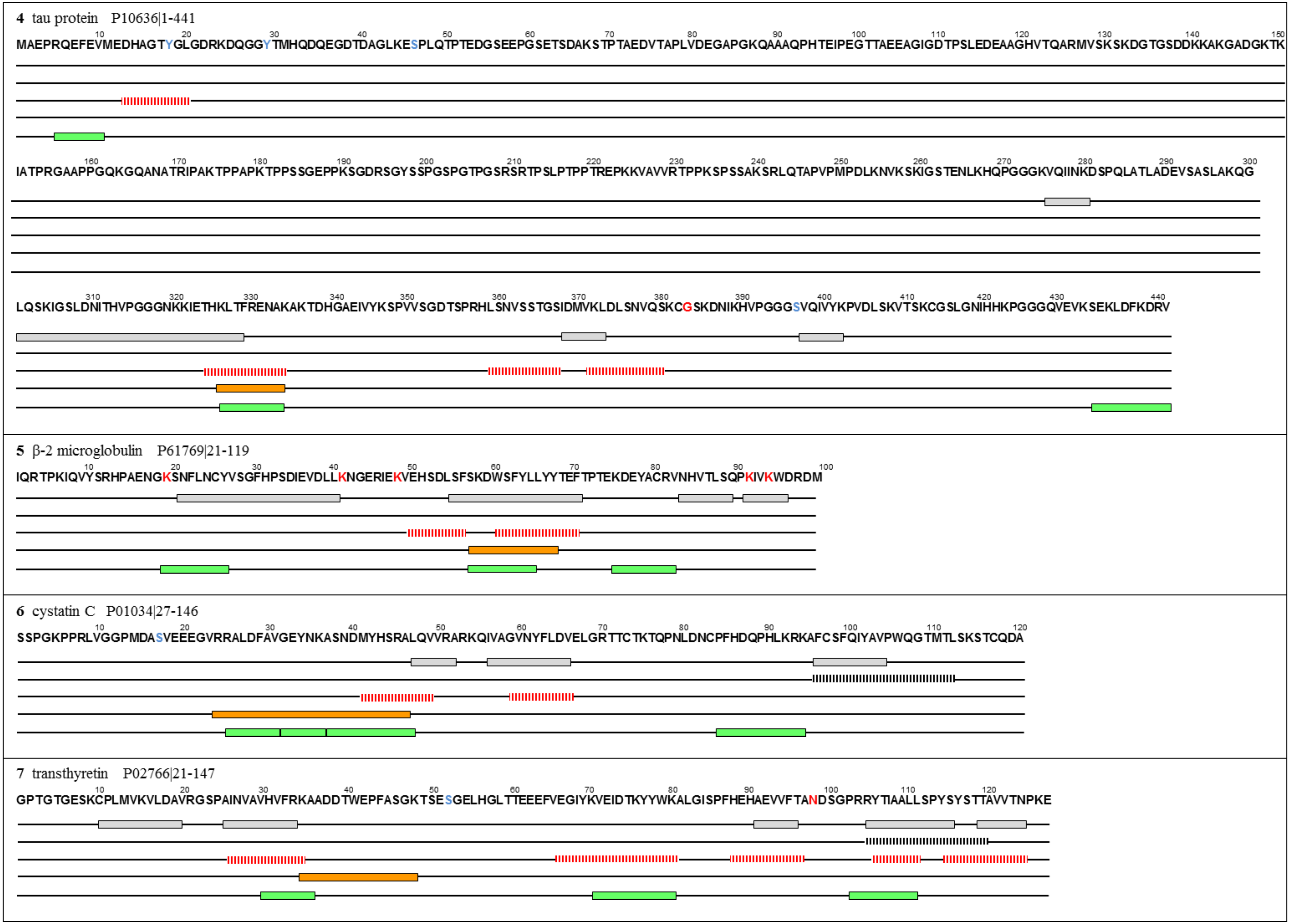

4 tau protein P10636|1-441
MAEPRQEFEVMEDHAGTYGLGDRKDQGGYTMHQDQEGDTDAGLKESPLQTPTEDGSEEPGSETSDAKSTPTAEDVTAPLVDEGAPGKQAAAQPHTEIPEGTTAEEAGIGDTPSLEDEAAGHVTQARMVSKSKDGTGSDDKKAKGADGKTK
IATPRGAAPPGQKGQANATRIPAKTPPAPKTPPSSGEPPKSGDRSGYSSPGSPGTPGSRSRTPSLPTPPTREPKKVAVVRTPPKSPSSAKSRLQTAPVPMPDLKNVKSKIGSTENLKHQPGGGKVQIINKDSPQLATLADEVSASLAKQG
LQSKIGSLDNITHVPGGGNKKIETHKLTFRENAKAKTDHGAEIVYKSPVVSGDTSPRHLSNVSSTGSIDMVKLDLSNVQSKCGSKDNIKHVPGGGSVQIVYKPVDLSKVTSKCGSLGNIHHKPGGGQVEVKSEKLDFKDRV
5 β-2 microglobulin P61769|21-119
IQRTPKIQVYSRHPAENGKSNFLNCYVSGFHPSDIEVDLLKNGERIEKVEHSDLSFSKDWSFYLLYYTEFTPTEKDEYACRVNHVTLSQPKIVKWDRDM
6 cystatin C P01034|27-146
SSPGKPPRLVGGPMDASVEEEGVRRALDFAVGEYNKASNDMYHSRALQVVRARKQIVAGVNYFLDVELGRTTCTKTQPNLDNCPFHDQPHLKRKAFCSFQIYAVPWQGTMTLSKSTCQDA
7 transthyretin P02766|21-147
GPTGTGESKCPLMVKVLDAVRGSPAINVAVHVFRKAADDTWEPFASGKTSESGELHGLTTEEEFVEGIYKVEIDTKYYWKALGISPFHEHAEVVFTANDSGPRRYTIAALLSPYSYSTTAVVTNPKE

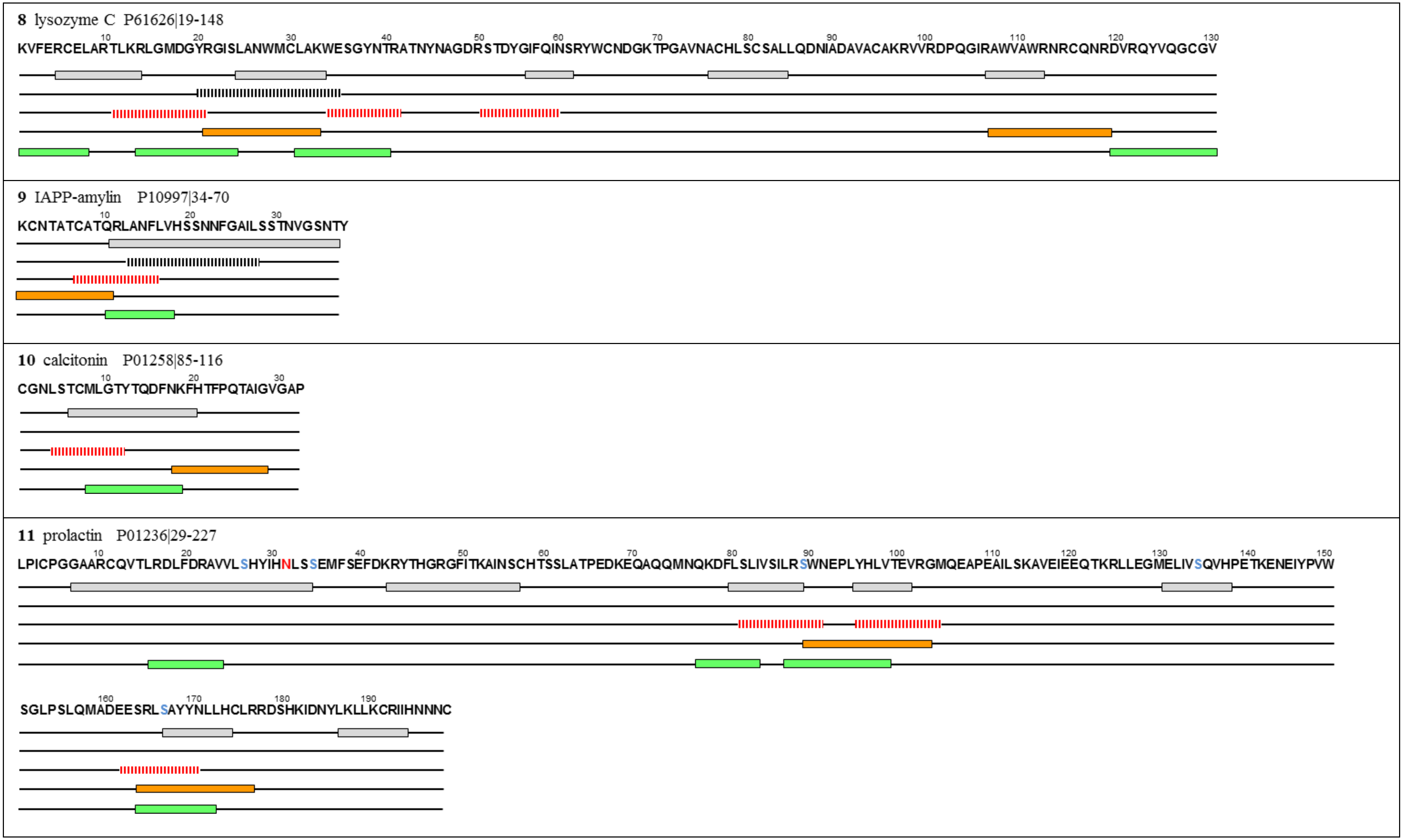
8 lysozyme C P61626|19-148
KVFERCELARTLKRLGMDGYRGISLANWMCLAKWESGYNTRATNYNAGDRSTDYGIFQINSRYWCNDGKTPGAVNACHLSCSALLQDNIADAVACAKRVVRDPQGIRAWVAWRNRCQNRDVRQYVQGCGV
9 IAPP-amylin P10997|34-70
KCNTATCATQRLANFLVHSSNNFGAILSSTNVGSNTY
10 calcitonin P01258|85-116
CGNLSTCMLGTYTQDFNKFHTFPQTAIGVGAP
11 prolactin P01236|29-227
LPICPGGAARCQVTLRDLFDRAVVLSHYIHNLSSEMFSEFDKRYTHGRGFITKAINSCHTSSLATPEDKEQAQQMNQKDFLSLIVSILRSWNEPLYHLVTEVRGMQEAPEAILSKAVEIEEQTKRLLEGMELIVSQVHPETKENEIYPVW
SGLPSLQMADEESRLSAYYNLLHCLRRDSHKIDNYLKLLKCRIIHNNNC

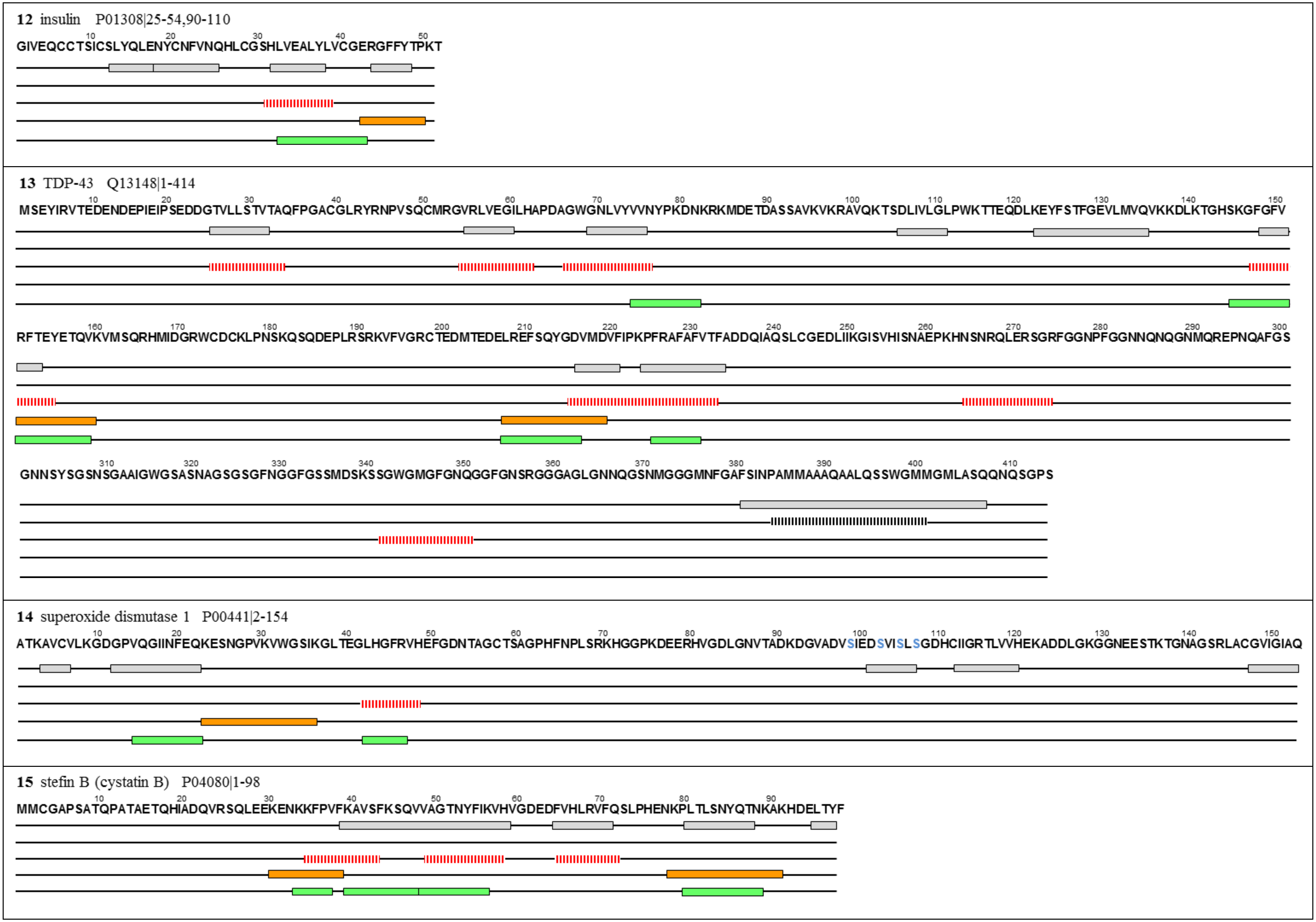

12 insulin P01308|25-54,90-110
GIVEQCCTSICSLYQLENYCNFVNQHLCGSHLVEALYLVCGERGFFYTPKT
13 TDP-43 Q13148|1-414
MSEYIRVTEDENDEPIEIPSEDDGTVLLSTVTAQFPGACGLRYRNPVSQCMRGVRLVEGILHAPDAGWGNLVYVVNYPKDNKRKMDETDASSAVKVKRAVQKTSDLIVLGLPWKTTEQDLKEYFSTFGEVLMVQVKKDLKTGHSKGFGFV
RFTEYETQVKVMSQRHMIDGRWCDCKLPNSKQSQDEPLRSRKVFVGRCTEDMTEDELREFSQYGDVMDVFIPKPFRAFAFVTFADDQIAQSLCGEDLIIKGISVHISNAEPKHNSNRQLERSGRFGGNPFGGNNQNQGNMQREPNQAFGS
GNNSYSGSNSGAAIGWGSASNAGSGSGFNGGFGSSMDSKSSGWGMGFGNQGGFGNSRGGGAGLGNNQGSNMGGGMNFGAFSINPAMMAAAQAALQSSWGMMGMLASQQNQSGPS
14 superoxide dismutase 1 P00441|2-154
ATKAVCVLKGDGPVQGIINFEQKESNGPVKVWGSIKGLTEGLHGFRVHEFGDNTAGCTSAGPHFNPLSRKHGGPKDEERHVGDLGNVTADKDGVADVSIEDSVISLSGDHCIIGRTLVVHEKADDLGKGGNEESTKTGNAGSRLACGVIGIAQ
15 stefin B (cystatin B) P04080|1-98
MMCGAPSATQPATAETQHIADQVRSQLEEKENKKFPVFKAVSFKSQVVAGTNYFIKVHVGDEDFVHLRVFQSLPHENKPLTLSNYQTNKAKHDELTYF

16 α-crystallin B chain P02511|1-175

MDIAIHHPWIRRPFFPFHSPSRLFDQFFGEHLLESDLFPTSTSLSPFYLRPPSFLRAPSWFDTGLSEMRLEKDRFSVNLDVKHFSPEELKVKVLGDVIEVHGKHEERQDEHGFISREFHRKYRIPADVDPLTITSSLSSDGVLTVNGPRK

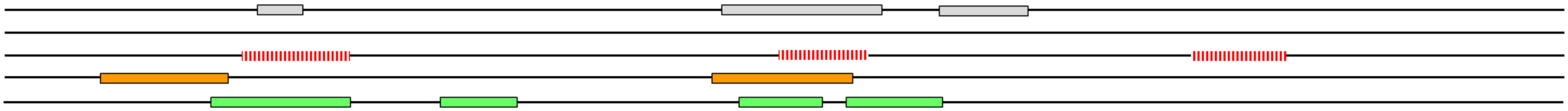

QVSGPKRTIPITREEKPAVTAAPKK

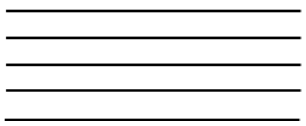

17 α-1-antichymotripsin P01011|24-423

HPNSPLDEENLTQENQDRGTHVDLGLASANVDFAFSLYKQLVLKAPDKNVIFSPLSISTALAFLSLGAHNTTLTEILKGLKFNLTETSEAEIHQSFQHLLRTLNQSSDELQLSMGNAMFVKEQLSLLDRFTEDAKRLYGSEAFATDFQDS

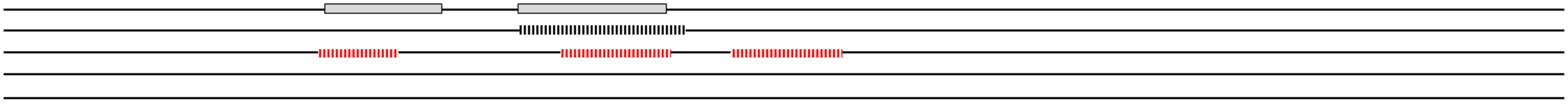

AAAKKLINDYVKNGTRGKITDLIKDLDSQTMMVLVNYIFFKAKWEMPFDPQDTHQSRFYLSKKKWVMVPMMSLHHLTIPYFRDEELSCTVVELKYTGNASALFILPDQDKMEEVEAMLLPETLKRWRDSLEFREIGELYLPKFSISRDYNLN

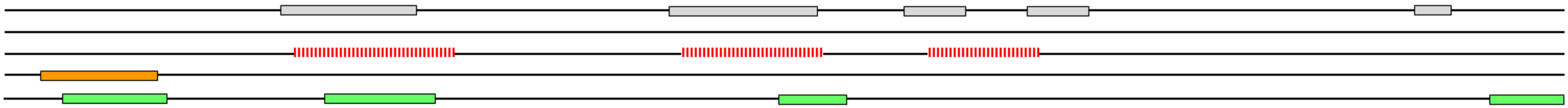

LNDILLQLGIEEAFTSKADLSGITGARNLAVSQVVHKAVLDVFEEGTEASAATAVKITLLSALVETRTIVRFNRPFLMIIVPTDTQNIFFMSKVTNPKQA

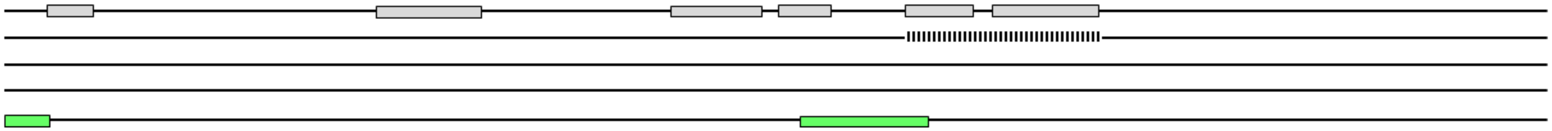

18 stefin A (cystatin A) P01040|1-98

MIPGGLSEAKPATPEIQEIVDKVKPQLEEKTNETYGKLEAVQYKTQVVAGTNYYIKVRAGDNKYMHLKVFKSLPGQNEDLVLTGYQVDKNKDDELTGF

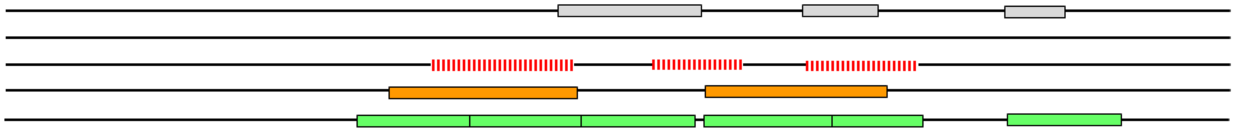

19 myglobin P02144|1-154

MGLSDGEWQLVLNVWGKVEADIPGHGQEVLIRLFKGHPETLEKFDKFKHLKSEDEMKASEDLKKHGATVLTALGGILKKKGHHEAEIKPLAQSHATKHKIPVKYLEFISECIIQVLQSKHPGDFGADAQGAMNKALELFRKDMASNYKELGFQG

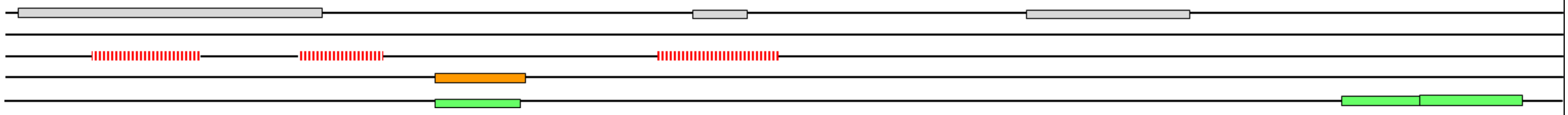

20 α-phosphatidylinositol 3-kinase P27986|1-84

MSAEGYQYRALYDYKKEREEDIDLHLGDILTVNKGSLVALGFSDGQEAKPEEIGWLNGYNETTGERGDFPGTYVEYIGRKKISP

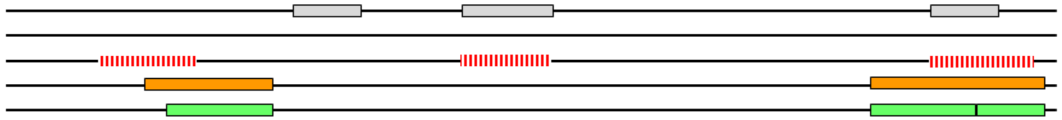

21 cathelicidin P49913|132-170

FALLGDFFRKSKEKIGKEFKRIVQRIKDFLRNLVPRTES

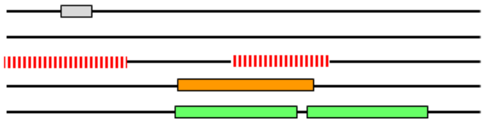

22 secretin P09683|28-54

HSDGTFTSELSRLREGARLQRLLQGLV

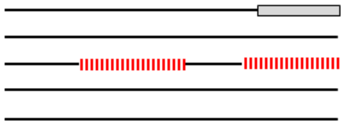

23 corticoliberin P06850|154-194

SEEPPISLDLTFHLLREVLEMARAEQLAQQAHSNRKLMEII

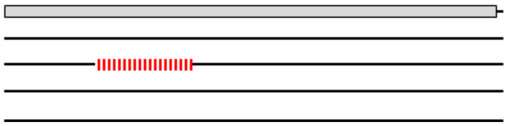

24 GIP – gastric inhibitory protein P09681|52-93

YAEGTFISDYSIAMDKIHQQDFVNWLLAQKGKKNDWKHNITQ

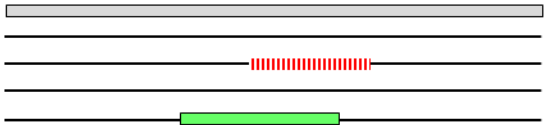

25 urocortin P55089|83-122

DNPSLSIDLTFHLLRTLLELARTQSQRERAEQNRIIFDSV

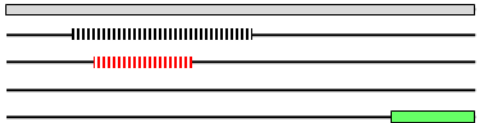

26 α-crystallin A chain P02489|1-173

MDVTIQHPWFKRTLGPFYPSRLFDQFFGEGLFEYDLLPFLSSTISPYYRQSLFRTVLDSGISEVRSDRDKFVIFLDVKHFSPEDLTVKVQDDFVEIHGKHNERQDDHGYISREFHRRYRLPSNVDQSALSCSLSADGMLTFCGPKIQTGL

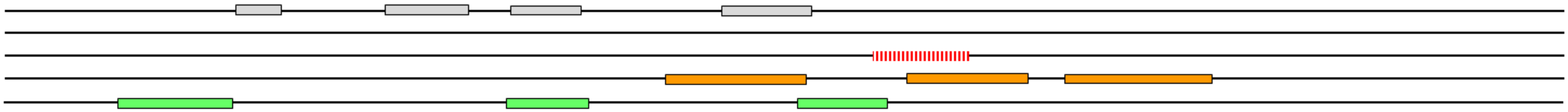

DATHAERAIPVSREEKPTSAPSS

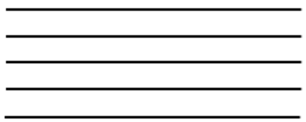

27 obestatin Q9UBU3|76-98

FNAPFDVGIKLSGVQYQQHSQAL

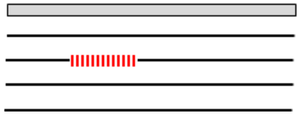

28 glucagon P01275|53-81

HSQGTFTSDYSKYLDSRRAQDFVQWLMNT

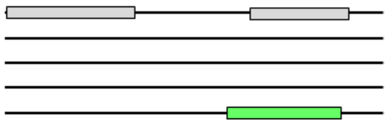

29 defensin-6 Q01524|69-100

AFTCHCRRSCYSTEYSYGTCTVMGINHRFCCL

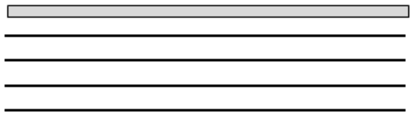

30 β-endorphin P01189|237-267

YGGFMTSEKSQTPLVTLFKNAIIKNAYKKGE

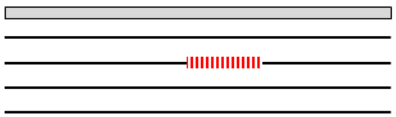

Table S6 Feature-based function prediction (FFPred) for membrane gene ontology domains for 30 amyloid-forming proteins (protein ID the same as in Table S1).

Gene ontology domain	Type of function prediction	1	2	3	4	5	6	7	8	9	10	11	12	13	14	15	16	17	18	19	20	21	22	23	24	25	26	27	28	29	30
	**Biological Process Predictions**																														
GO:0055085	TM transport	x							x	x	x									x								x	x	x	
GO:0034220	ion TM transport	x								x	x		x																x	x	
GO:0098655	cation TM transport	x								x	x																	x	x	x	
	**Molecular Function Predictions**																														
GO:0022857	TM transporter activity	x								x	x										x							x	x	x	x
GO:0015075	ion TM transporter activity	x								x	x										x				x	x		x	x	x	
GO:0008324	cation TM transporter activity	x								x	x										x							x	x	x	x
GO:0022891	substrate-specific TM transporter activity								x	x						x												x			
GO:0046873	metal ion TM transporter activity	x								x	x																				
GO:0005216	ion channel activity	x													x				x		x							x		x	
GO:0022890	inorganic cation TM transporter activity	x								x																					
	**Cellular Component Predictions**																														
GO:0016020	membrane	x	x	x	x	x	x	x	x	x	x	x	x		x	x	x			x	x	x	x	x	x	x	x	x	x	x	x
GO:0016021	integral component of membrane	x		x						x	x		x										x			x			x	x	
GO:0012505	endomembrane system	x		x		x	x			x		x																			
GO:0005886	plasma membrane	x	x	x	x	x	x	x		x	x				x						x										
GO:0031982	vesicle	x		x		x	x	x		x	x	x			x	x	x		x	x	x	x									
GO:0031988	membrane-bounded vesicle			x		x	x	x	x	x	x	x			x	x	x		x	x		x					x	x	x	x	x
GO:0031224	intrinsic component of membrane	x		x			x		x	x	x		x										x			x		x	x	x	x
GO:0005887	integral component of plasma membrane												x			x															
GO:0031226	intrinsic component of plasma membrane												x																		
GO:1902495	TM transporter complex	x																													
